# Systematic evaluation of B-cell clonal family inference approaches

**DOI:** 10.1186/s12865-024-00600-8

**Published:** 2024-02-08

**Authors:** Daria Balashova, Barbera D. C. van Schaik, Maria Stratigopoulou, Jeroen E. J. Guikema, Tom G. Caniels, Mathieu Claireaux, Marit J. van Gils, Anne Musters, Dornatien C. Anang, Niek de Vries, Victor Greiff, Antoine H. C. van Kampen

**Affiliations:** 1 Amsterdam UMC location University of Amsterdam, Epidemiology and Data Science, Meibergdreef 9, Amsterdam, Netherlands; 2 Amsterdam Public Health, Methodology, Amsterdam, The Netherlands; 3 Amsterdam Infection and Immunity, Inflammatory Diseases, Amsterdam, The Netherlands; 4 Cancer Center Amsterdam, Amsterdam, The Netherlands; 5 Amsterdam UMC location University of Amsterdam, Medical Microbiology and Infection Prevention, Meibergdreef 9, Amsterdam, Netherlands; 6 Amsterdam Infection and Immunity, Infectious Diseases, Amsterdam, The Netherlands; 7 Amsterdam UMC location University of Amsterdam, Experimental Immunology, Meibergdreef 9, Amsterdam, Netherlands; 8 Amsterdam Rheumatology & Immunology Center, Amsterdam, The Netherlands; 9 Amsterdam UMC location University of Amsterdam, Pathology, Lymphoma and Myeloma Center Amsterdam, Meibergdreef 9, Amsterdam, Netherlands; 10 Department of Immunology, University of Oslo and Oslo University Hospital, Oslo, Norway; 11 Biosystems Data Analysis, Swammerdam Institute for Life Sciences, University of Amsterdam, Amsterdam, The Netherlands

**Keywords:** B-cell receptor repertoire, B-cell clonal family partitioning, AIRR-seq data, AIRR-seq data simulation, B-cell shared clonal families

## Abstract

The reconstruction of clonal families (CFs) in B-cell receptor (BCR) repertoire analysis is a crucial step to understand the adaptive immune system and how it responds to antigens. The BCR repertoire of an individual is formed throughout life and is diverse due to several factors such as gene recombination and somatic hypermutation. The use of Adaptive Immune Receptor Repertoire sequencing (AIRR-seq) using next generation sequencing enabled the generation of full BCR repertoires that also include rare CFs. The reconstruction of CFs from AIRR-seq data is challenging and several approaches have been developed to solve this problem. Currently, most methods use the heavy chain (HC) only, as it is more variable than the light chain (LC). CF reconstruction options include the definition of appropriate sequence similarity measures, the use of shared mutations among sequences, and the possibility of reconstruction without preliminary clustering based on V- and J-gene annotation. In this study, we aimed to systematically evaluate different approaches for CF reconstruction and to determine their impact on various outcome measures such as the number of CFs derived, the size of the CFs, and the accuracy of the reconstruction. The methods were compared to each other and to a method that groups sequences based on identical junction sequences and another method that only determines subclones. We found that after accounting for data set variability, in particular sequencing depth and mutation load, the reconstruction approach has an impact on part of the outcome measures, including the number of CFs. Simulations indicate that unique junctions and subclones should not be used as substitutes for CF and that more complex methods do not outperform simpler methods. Also, we conclude that different approaches differ in their ability to correctly reconstruct CFs when not considering the LC and to identify shared CFs. The results showed the effect of different approaches on the reconstruction of CFs and highlighted the importance of choosing an appropriate method.

**Supplementary Information:**

The online version contains supplementary material available at 10.1186/s12865-024-00600-8.

## Introduction

B cells are a type of lymphocytes that play an important role in the adaptive immune system. These cells express B-cell receptors (BCR) comprising two identical heavy chains (HC) and two identical light chains (LC) that allow the B cell to bind to an antigen. An individual’s repertoire of B cells is formed throughout life partly as a result of immune responses to pathogenic antigens. A huge diversity of BCRs can theoretically be achieved, and is estimated to be 10^18^ receptors based on theoretical combinatorial calculations and several factors limit the actual size to about 10^15^ for the naive repertoire [1]. Initial BCR variability occurs due to somatic recombination during which the HC is formed by a random combination of V, D and J genes, while the LC is formed by V and J genes [2]. Further variability is imposed by the recombination process due to additional nucleotide insertions and deletions at the junctions of genes (junctional diversity). A second level of diversity is the result of the random pairing of HCs and LCs resulting in a naive, antigen inexperienced, B cell. During an affinity maturation additional variability is introduced by somatic hypermutation (SHM) in the germinal center (GC) [3]. The GC reaction facilitates affinity maturation of the BCR through iterative cycles of proliferation and somatic mutation. This leads to expanded clones with high affinity BCRs. Using Adaptive Immune Receptor Repertoire sequencing (AIRR-seq), it has become possible to determine the BCR repertoire of a sample. This high-throughput sequencing approach leads to one or more sequences for each unique BCR in the sample. One critical step is to determine which (somatically mutated) sequences belong to the same clonal family (CF), which represent all B cells (and thus BCRs) originating from the same unmutated common ancestor (germline sequence) [4]. Each CF comprises identical or similar V(D)J sequences that differ only as the result of SHM or, in rare cases, by V gene replacement [5]. The size of each CF is determined by its number of sequences and can be used to identify dominant (highly expanded) CFs that are hypothesized to be the main participants in an immune response. Each CF may include multiple subclones, which are defined as all cells with identical BCRs. The reconstruction of CFs and determination of their frequencies from AIRR-seq data is a crucial step to facilitate further analyses and interpretation of the measured BCR repertoires. First common steps in the analyses involve, for example, the establishment of the number and size of CFs within a sample, the number of dominant clones, and repertoire diversity [6]. Subsequently, dedicated analyses are performed to address specific biological questions. For example, AIRR-seq sequencing has been used for the identification of shared CFs among individuals [7–9], the characterization of abnormal immune repertoires in primary immunodeficiencies [10], the identification of stereotyped BCRs in chronic lymphocytic leukemia patients [11], or the analysis of anti-drug antibodies development in multiple sclerosis patients [12].

However, the reconstruction of CFs is not without challenges and a fully correct reconstruction is an unsolved problem. Consequently, analysis and interpretation of BCR repertoires may depend on the choice of a specific reconstruction approach but this has never been extensively investigated. A range of approaches to infer CFs from AIRR-seq data are available [13–17]. One problem is the difficulty in accurately reconstructing the germline sequence, which would facilitate the reconstruction process. This is mainly caused by the high variability in the CDR3 region that contains the D gene, and the high similarity between several V and J genes. Most current methods only use the HC because it is the most variable chain [2] and because pairing with the LC proved difficult. It was recently shown that HC-based CFs are accurate for over 80% if the LC is not incorporated in the reconstruction process [18], while [19] claims that this accuracy might be lower for larger samples. In any case, leaving out the LC is a potential source of error that may experimentally be addressed by single cell RNA sequencing [20]. Other challenges involve the definition of appropriate similarity measures to decide if sequences originate from the same germline sequence, the exploitation of full-length sequence variability instead of focusing on the CDR3 region, the use of both shared and unique mutations among sequences, and approaching reconstruction without a preliminary clustering step, based on V- and J-gene annotation. The methods referred to above approach these challenges in different ways.

Evaluation of methodology for CF reconstruction is important because it helps to identify potential biases, errors, or limitations related to study design (e.g., patient diversity, sequencing depth), which could affect the validity and reliability of the findings. For example, phylogenetic analyses to reconstruct B-cell lineages critically rely on the quality of the CFs [21–23].

We aimed to systematically evaluate different approaches for CF reconstruction and selected eight methods to determine the effect on several outcome measures such as the number of CFs derived with each of these methods. We complemented these methods with a method that just groups sequences based on identical junction sequences, and with a method that only determines the subclones (identical V and J gene, and identical junction sequence). In addition, we aimed to show how differences stemming from these methods affect the identification of shared CFs, as one example of a more downstream analysis. In addition, we determined the LC concordance for each of these methods based on two single cell repertoire datasets. Since the performance of different methods may be dependent on dataset characteristics such as sequencing depth and mutation load, we applied each method to eight different datasets. Since for none of the datasets we know the true CF structure, a comparison only reveals differences between the methods but cannot identify the best performing method [24]. Therefore, we also applied each method to simulated datasets for which the CFs are known by definition.

We showed that most approaches for CF reconstruction perform similarly, although Change-O [13] best reproduces the true CF structure but does not produce CFs with a higher LC concordance. SCOPer [15, 16] and the alignment free method [17] seem to perform less well. We also show that clustering unique junction sequences or subclones cannot be used as surrogates for real CFs, although the outcome measures for these two approaches deviate much less than expected from other reconstruction approaches. In general, more sophisticated methods do not outperform more straightforward approaches to cluster sequences into CFs. We also show that sequencing depth and mutation load both affect the reconstruction process. Finally, we show that the number of shared (dominant) CFs identified varies between the approaches but given the limited amount of data we cannot establish if differences between the approaches are statistically or biologically significant. In general, our results show that there is room to further improve methods for CF reconstruction.

## Materials and methods

### Clonal family inference approaches evaluation

**Fig. 1:**
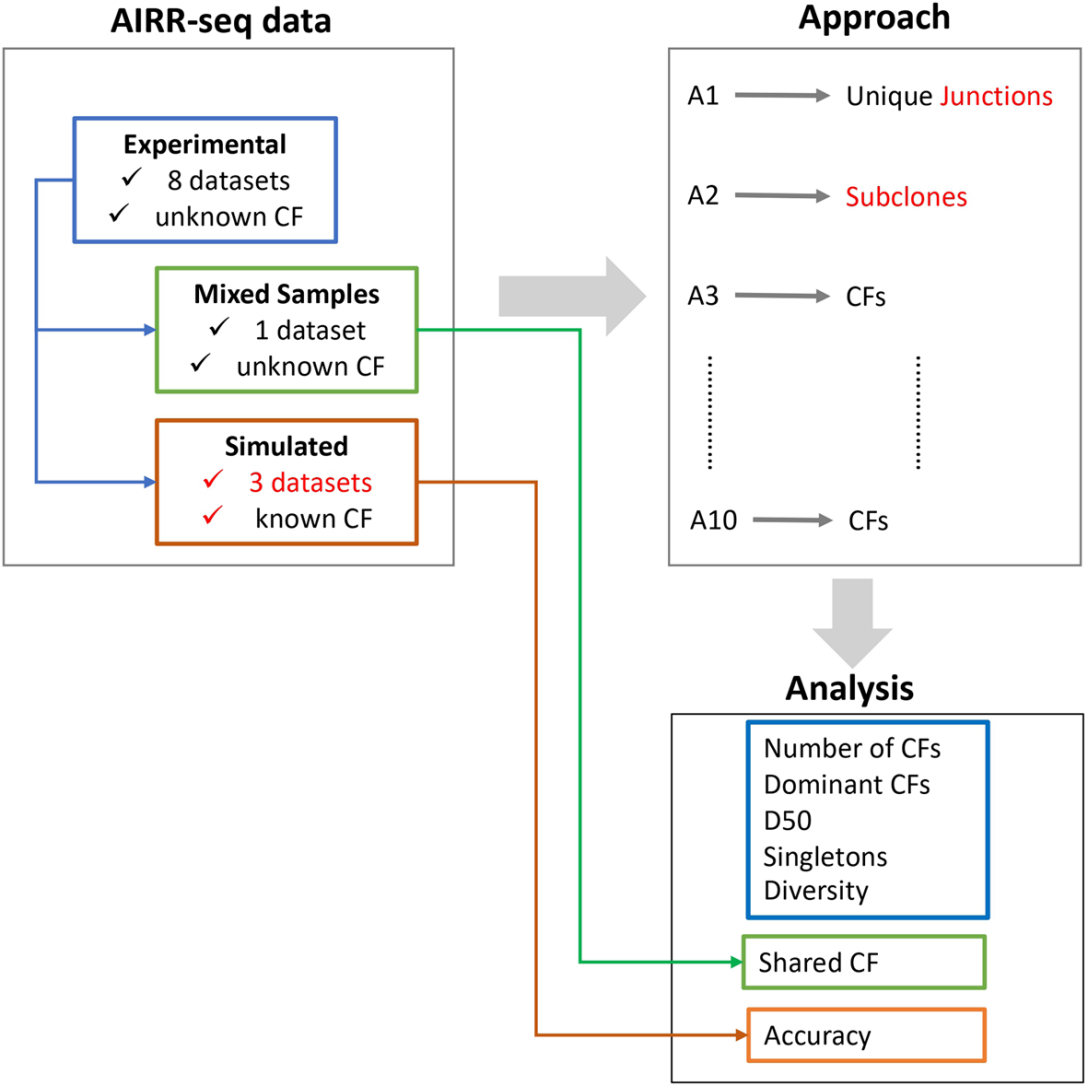
Study design. Eight CF reconstruction approaches (A3 – A10) were applied to eight AIRR-seq datasets, one mixed dataset, and three simulated datasets. In addition, we determined the number of unique junctions (A1) and subclones (A2) from each dataset. The mixed sample was generated from the experimental data. Our simulation approach used the experimental data as input to derive CFs. Results from all 10 approaches were analyzed. For the mixed and simulated samples, we also determined the number of shared clones and accuracy respectively

We evaluated eight approaches (A3 – A10) for the reconstruction of CFs (Fig. 1**;** Table 1). None of these methods aims to reconstruct the D genes, since they are too short and variable. Instead, we consider the V and J genes in combination with the junction, which comprises the CDR3 sequence including its two anchors CYS104 and PHE/TRP118 [25]. The CDR3 is the most variable Ig region and directly involved in antigen binding [2, 26]. For comparison we also determined the number of unique junction sequences (A1) and number of subclones (A2; identical V and J gene and identical junction). These 10 approaches were applied to three selected samples from eight different AIRR-seq datasets (Table 2). Since the true number of CFs is unknown for the experimental data, we also simulated three datasets to determine the accuracy of each method. The CFs derived by each method were further analyzed to determine the number of CFs, the CF size, the number of dominant CFs, D50, and the diversity. BCR clones with a frequency above 0.5% were defined as dominant clones [27]. D50 was defined as the number of CFs that account for 50% of sequences in the sample. Singletons are CFs that consist of one sequence. Several diversity measures are used in repertoire sequencing studies [6, 28]. We selected the Shannon index, which does not bias rare or common CFs, and the Gini-Simpson index that emphasizes the common CFs [29]. The Shannon and Simpson diversity indices were derived from the Hill-based diversity $^{\alpha }D(A)$ index, which is defined as

$$^{\boldsymbol{\alpha}}\boldsymbol{D}\left(\boldsymbol{f}\right)={\left(\sum_{\boldsymbol{i}=\textbf{1}}^{\boldsymbol{n}}{{\boldsymbol{f}}_{\boldsymbol{i}}}^{\boldsymbol{\alpha}}\right)}^{\frac{\textbf{1}}{\textbf{1}-\boldsymbol{\alpha}}}$$

where *f* is the clonal frequency distribution with *f*_*i*_ being the frequency of each CF defined by the number of BCR sequences it includes, and *n* being the total number of CFs. From this we obtain the Shannon index and Simpson reciprocal diversity indices [29]:

**Table 1:** Clonal family reconstruction approaches. NT: nucleotide; AA: amino acid

	Approach	VJ Partioning	Region	Sequence Type	Identical Junction Length	Similarity Measure	Sequence Clustering
**A1**	Unique junction (AA)	No	Junction	AA	Yes	Exact match	Dissimilarity = 0%
**A2**	Subclone (AA)	Yes	Junction	AA	Yes	Exact match	Dissimilarity = 0%
**A3**	Absolute threshold (AA)	Yes	Junction	AA	Yes	Hamming Distance between junction regions	Dissimilarity <= 1 AA (absolute threshold)
**A4**	Relative threshold (AA)	Yes	Junction	AA	Yes	Length normalized Hamming Distance between junction regions	Dissimilarity <=15% (relative threshold)
**A5**	Relative threshold (NT)	Yes	Junction	NT	Yes	Length normalized Hamming Distance between junction regions	Dissimilarity <=15% (relative threshold)
**A6**	Change-O	Yes	Junction	NT	Yes	Length normalized Hamming Distance between junction regions	Sample-based dissimilarity threshold based on bimodal distance-to-nearest distribution
**A7**	SCOPer (junction)	Yes	Junction	NT	Yes	Kernel matrix (distance based on junction)	Unsupervised spectral clustering
**A8**	SCOPer (shared)	Yes	Junction + VJ sequence	NT	Yes	Kernel matrix (distance based on junction + shared mutations in VJ)	Unsupervised spectral clustering
**A9**	Partis	Yes	Full sequence	NT	No	Likelihood ratio to decide if two sequence (sets) were derived from same ancestor, and Hamming distance between reconstructed germline sequences.	Hamming Dissimilarity <=1.5% OR Likelihood ratio < = variable threshold
**A10**	Alignment free	No	Full sequence	NT	No	Cosine distance calculated from the tf-idf statistic.	Automatic clonal distance threshold determination by negation, fraction of the distances to negation sequences threshold = 10%

**Table 2:** Selected AIRR-seq datasets. For each of eight datasets we used three samples. Ig: immunoglobulin. GC: germinal center; PB: peripheral blood; SF: synovial fluid; ST: synovial tissue; HC: heavy chain; LC: light chain; sc: single cell

Dataset	Source	Number of patients, samples	Tissue	Source	Ig chain	Mutation load (%)	Sequencing depth
D1	Chronic sialadenitis [40]	1, 3	single GC	DNA	HC	3.1	144,626
						2.5	157,020
						3.3	125,092
D2	Rheumatoid arthritis [41]	3,3	PB	RNA	HC	2.7	88,492
						1.4	52,876
						1.3	54,075
D3	Rheumatoid arthritis [41]	1,3	ST/SF	RNA	HC	5.5	88,075
						1.7	81,566
						1.6	109,298
D4	Healthy donor [42]	3,3	PB	scRNA	Paired HC/LC	1.3	962
						0.2	1118
						0.4	1236
D5	Healthy donor [43]	3,3	PB	scRNA	Paired HC/LC	3.6	452,627
						4.0	540,914
						4.1	115,728
D6	HIV infected [44]	3,3	PB	RNA	HC	3.7	201,048
						4.5	188,309
						5.7	239,189
D7	HIV uninfected [44]	3,3	PB	RNA	HC	3.6	147,340
						5.0	155,456
						3.2	146,121
D8	Crohn’s disease [36]	3,3	PB	RNA	HC	5.2	85,841
						4.0	92,112
						4.5	131,725

*Shannon  index*(*f*) = ln^1^*D*(*f*)

$$Simpson\kern0.34em reciprocal\kern0.17em index(f)=\frac{1}{^2D(f)}$$

We report the Gini-Simpson = (1 – Simpson reciprocal index) [30], which ranges between 0 (low diversity) and 1 (high diversity), and can be interpreted as the probability that two randomly selected sequences belong to different CFs. The Shannon index reflects the uncertainty about the identification of sequences in the repertoire (measured in bits [31];) and ranges between its maximum value of *ln* (CF) (high diversity) and 0 (low diversity) [32].

### Clonal family inference approaches

**Fig. 2:**
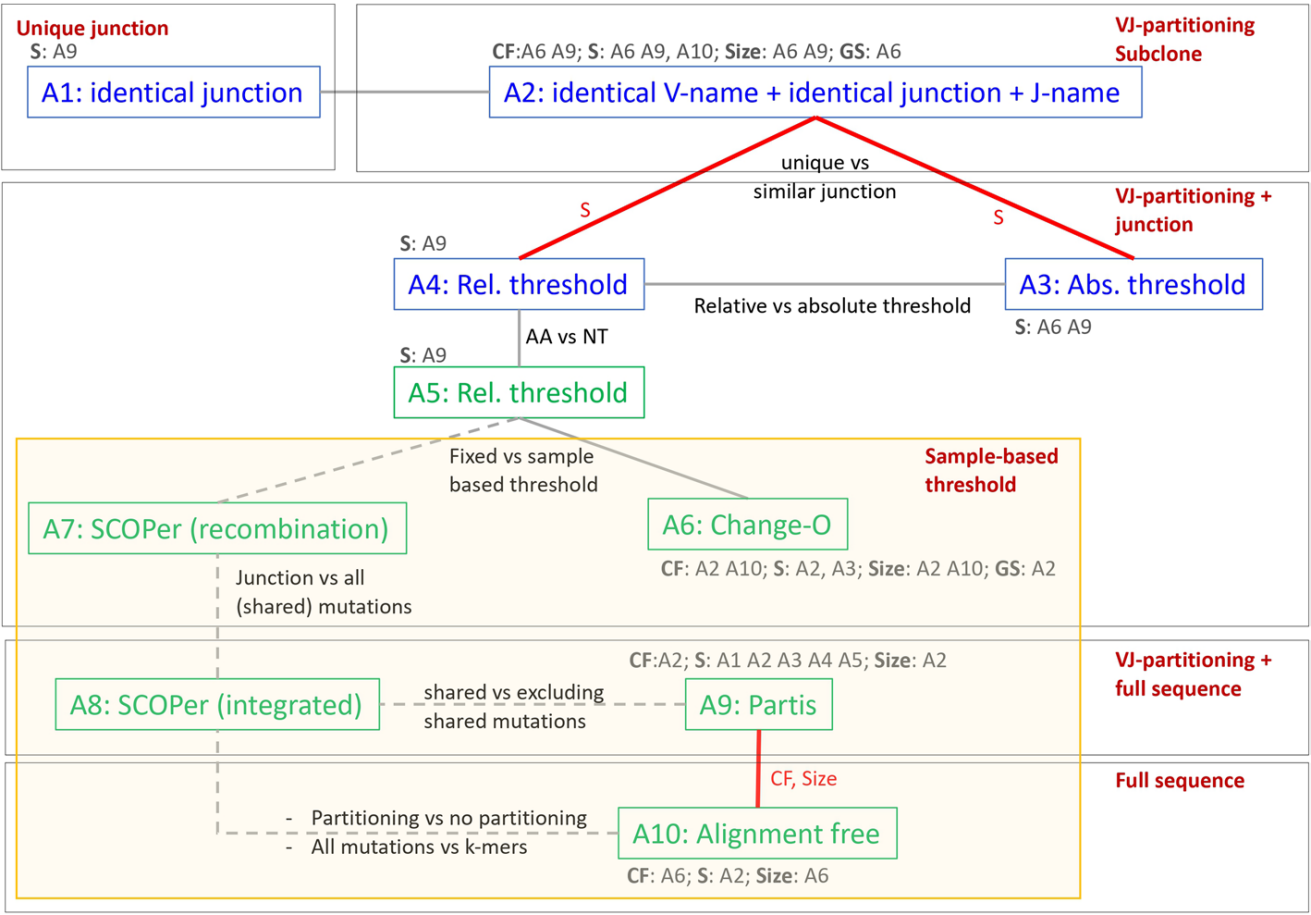
CF inference approaches. Grey lines connecting boxes represent specific comparisons of interest. Red lines and text indicate the significant comparisons of interest. Dashed grey lines denote comparisons that we could not carry out. A1 and A2 don’t represent CFs but unique junction sequences and subclones respectively. Green boxes: nucleotide sequences; Blue boxes: amino acid sequences; Grey text: significant comparisons between non-connected approaches for CFs, singletons (S), mean CF size (Size), and Gini-Simpson (GS)

Figure 2 and Table 1 show the eight selected CF reconstruction approaches that we evaluated in our study. The connected approaches in the figure indicate specific comparisons of interest, such as using a fixed (A5) versus a sample-based (A6) similarity threshold. We classified the approaches according to (i) V/J partitioning of the sequences to ensure all sequences of a CF use the same V/J gene, (ii) sequence type (nucleotides or amino acids) and (iii) use of the full versus the junction sequence. Note that for consistency, we consider junction sequences instead of the CDR3 sequence, although in [15] it was suggested that SCOPer (A7, A8) produces slightly better results when using the CDR3 sequence.

Approach A1 and A2 do not reconstruct CFs but were included as a reference. Approach A1 clusters identical junction amino acid sequences, while Approach A2 identifies subclones defined as sequences with identical V- and J-gene names, and an identical junction amino acid sequence. A1 is commonly used in papers for BCR repertoire analysis [6, 33, 34]. Approaches A3, A4, and A5 reconstruct CF by grouping sequences with identical V- and J-gene names and similar junction sequences of equal length. For these methods, junction similarity is based on the Hamming distance between the nucleotide (A5) or amino acid (A3, A4) sequence, and sequences with a similarity above the fixed absolute (A3) or relative (A4, A5) thresholds are grouped together. The relative threshold ensures 85% sequence similarity within each CF, while the absolute threshold allowed a maximum of one amino acid between the sequences. Comparison of A4 and A5 allows to establish an effect of the sequence representation. To group sequences into CFs with approach A3, A4, or A5 we first construct a fully connected graph of sequences with each edge representing the similarity between a pair of sequences. Subsequently, we remove all edges representing similarities less than the defined threshold. Variations on approaches with a fixed threshold have been presented previously in other studies. For example, an approach similar to A3 but using an absolute threshold of one nucleotide was used in [35, 36]. Approach A4 has been used by Soto and co-workers but using a 20% similarity threshold [34], while an approach similar to A5 but without V/J partitioning is presented in [37].

A main drawback of approaches A3, A4, A5 is the use of an arbitrary similarity threshold that is not based on the data itself and, therefore, may not result in the best possible reconstruction of the CFs. Therefore, other approaches (A6 – A10) have been developed that aim to determine this threshold from the data. Change-O (A6; Nouri, 2018) aims to determine the optimal threshold on a per-sample basis by fitting a smoothed density to the normalized Hamming distances between all sequences for the sample.

Approaches A2 to A9 suffer from possible sequence alignment errors made during the V/J gene assignment during which the repertoire sequences are matched against a reference database using, for example, IgBlast [38] and the IMGT germline V, D, J gene databases [25]. In addition, approaches A1-A6 do not consider the full VDJ sequence of the receptor, but only consider junction similarity. Consequently, these approaches neglect somatic mutations in the V and J genes and this potentially leads to the merging of CFs because the sequence similarity is estimated too high. Recently, a new method (A7, A8; SCOPer) was developed that comprises two models to reconstruct CFs [15, 16]. Both models start with a VJ-partitioning. The recombination-based model (A7) only considers the Hamming distance between pairs of junction sequences, while the second integrated model (A8) combines the recombination-based model with an SHM-based distance that is based on total and shared number of mutations in pairs of V/J sequences while accounting for hot/cold spots based on the S5F targeting model [39]. For both models the final similarity between all sequence pairs is calculated from a Gaussian kernel to allow the local level of similarity to vary in a fully connected graph of all sequences. Subsequently, a spectral decomposition procedure is used to determine the number of CFs, followed by k-means clustering to reconstruct the CFs. Comparison of SCOPer to other approaches allows us to establish the added value of considering (shared) mutations in the V and J sequence.

Another method that utilizes the full sequence is partis (A9), which is based on a multi-hidden Markov model (HMM) framework [19]. The HMM framework is used to define a likelihood ratio to determine if two sequences (or sequence sets) come from a single rearrangement event and, therefore, should be merged into a single CF. Consequently, Partis considers all substitution mutations in the V-Junction-J sequence. The likelihood ratio is used as the distance measure for agglomerative clustering using a variable likelihood ratio threshold, based on the cluster size, to decide if clusters/sequences should be merged. To accelerate computations, the normalized Hamming distance between inferred germline sequences from both clusters/sequences is used to decide if clusters/sequences should be merged (<=0.015) or should not be merged (> = 0.08) without calculating the likelihood ratio.

Finally, an approach based on natural language processing (A10) was introduced and uses the full sequence and doesn’t require VJ-partitioning [17]. The approach uses the term frequency inverse document frequency (tf-idf) weighted k-mer representation to utilize the full receptor sequence. Automatic clonal distance threshold determination is accomplished by negation, i.e., using sequences from a different sample, which are supposed to be at greater distances than sequences within a sample. The tf-idf emphasizes the rare and meaningful sequence motifs and reduces the influence of common motifs. A cosine distance calculated from the tf-idf is then used to determine the sequences belonging to a CF. This method doesn’t require a sequence alignment to assign the V and J genes and doesn’t require junction sequences to be of the same length.

### Experimental AIRR-seq data

We reasoned that the performance of CF reconstruction methods might depend on characteristics of the dataset to which it is applied such as sequencing depth, mutational load, DNA or RNA sequencing, and sequencing of HC or paired HC/LC. Therefore, for the comparison of the different approaches, we selected three samples from eight different AIRR-seq datasets (Table 2). The mutation load was presented as the percentage of mutated nucleotides, and calculated by averaging over all BCR sequences the number of differences between in the V and J gene sequences and the corresponding IMGT germline sequences [25] divided by the length of the V or J gene sequence, i.e., (number of mutations in V aligned segment + number of mutations in V aligned segment) / (length of V aligned germline segment + length of J aligned germline segment). This excludes the mutations in the most variable CDR3 region of the sequence since it is virtually impossible to reliably reconstruct the germline D gene and non-templated nucleotides and, therefore, our percentage underestimates the number of acquired mutations. Moreover, we assume that each difference observed in the V and J genes is a somatic mutation and not a PCR or sequencing error. We also disregarded germline gene polymorphisms at the V/J partitioning stage used in approaches A2-A8, since their identification is difficult and the alleles listed by IMGT are incomplete. This might lead to sequences being misclassified due to incorrect annotation of the V and J and affect the CF reconstruction.

Dataset D1 comprises a DNA-based repertoire from single GCs isolated from a human lymph node from a tonsil from a patient with chronic sialadenitis [40]. We included these samples because we expected that repertoire data from GCs is more homogeneous (fewer and more similar clones) compared to tissue or blood samples. In addition, we included D1 (and D3) because we required samples derived from a single patient for the shared CF analysis (see below). Dataset D2 and D3 comprises bulk RNA-seq based repertoires measured from peripheral blood, synovial tissue and synovial fluid samples obtained from three rheumatoid arthritis (RA) patients [41]. Datasets D4 [42] and D5 [43] represent single cell (sc)RNA-seq based repertoires from healthy donors. We included these repertoires to investigate the consistency of clonal inference results based on HC only versus using the paired HC and LC. Note that D4 has a very low sequencing depth, while D5 has the highest sequencing depth. Datasets D6 and D7 represent bulk RNA-seq based repertoire obtained from HIV infected and non-infected patients [44]. We selected three samples with the highest mutation load from the HIV infected patients and randomly selected three samples from the non-infected individuals. We expected the mutation load of the HIV infected patients to be higher in comparison with the other samples we included in our study, but Table 2 shows that the mutation load in the V/J genes is comparable (or even less) compared to several other samples. The differences between the HIV infected patients and the other samples might be more pronounced if the CDR3 mutations could have been included. Dataset D8 is a selection of three RNA-seq based repertoires from Crohn’s disease patients. These samples were taken from a larger study that investigated pathological mechanisms in autoimmune-mediated disease [36].

We pre-processed the raw sequencing data (FASTQ/FASTA files) of each of the experimental AIRR-seq datasets used in our study. In short, we removed the primer sequences and, subsequently, data we identified the V and J genes and junction region for each sequence using IgBlast [38] version 1.17.1 using the most similar IgBlast hit.

### Simulated data

For simulations we used samples from D1, D2 and D3 datasets. We simulated three repertoire datasets (D10 – D12) to determine which reconstruction approach closest resembles the true number of CFs (Supplementary Fig. 1). This allows us to calculate the accuracy of each of the approaches. To simulate data, we use subclones and CFs from all approaches because we need fair comparison (thus, each of approaches represented equally in simulations) of their accuracy, similar to how we compare the results of approaches applied to experimental data.

Our goal is to simulate data that accurately reflects the characteristics of actual experimental repertoires. Therefore, each individual simulated sample was based on a single sample selected from dataset D1, D2 or D3 (Table 2). We generated six simulated datasets (S28 – S33 in Supplementary Table 1 (S3)) by using two samples from each of these datasets. The input for the simulation was provided by the unique junction sequences, subclones, and CFs resulting from application of approaches A1, A2 and A3-A10 respectively to the selected dataset. Our simulation approach is an integration of ImmuneSim [45], Alakazam [13] and SHazaM [39] packages. We used the default (except where specified below) parameters for these methods which can be found in the documentation of commands immuneSIM, buildPhylipLineage and shmulateTree of ImmuneSim, Alakazam and SHazaM packages respectively. The simulation proceeded along the following steps:

- **Step 1**. For the chosen sample we randomly selected 10% of the unique junctions/subclones/CFs obtained from the application of each of the 10 approaches applied to the selected sample;
- **Step 2**. We assigned the V/J-gene names to each sequence using IgBlast. For sequences that were assigned to multiple V/J-gene names we used the most frequent V/J gene;
- **Step 3**. We simulated VDJ recombination by using the V/J usage frequencies observed in the set of selected sequences (step 2), and the frequency usage for the D genes provided by ImmuneSim [45];
- **Step 4**. For each CF that was selected in Step 1, we reconstructed the B-cell lineage tree using the maximum parsimony method from Alakazam [13]. To facilitate this, we used only sequences of identical length, i.e., we removed the sequences that were different from the most common sequence length of a CF. The resulting lineage reflected all sequences that make up a CF and the SHM’s imposed on the B-cell receptors during clonal expansion. Each node in this tree corresponded to a subclone;
- **Step 5.** We randomly selected a germline sequence obtained from ImmuneSim (Step 3) to serve as the unmutated root of the B-cell lineage obtained in Step 4. Subsequently, using this germline sequence and the B-cell lineage topology, we use SHazaM [39] to create a new B-cell lineage that leaves the topology intact but imposes a new set of mutations. This provided us with a set of simulated sequences. The imposed mutations were based on the HH_S5F (Human heavy chain, silent, 5-mer, functional targeting model) described in [39].

### Evaluation of the approach accuracy from simulated data

To obtain a measure of the accuracy of each reconstruction approach we compare the simulated CFs (ground truth) to the CFs obtained from each individual approach (A1 – A10). To facilitate this comparison, we represent each simulated and reconstructed CF as a graph of connected sequences (edges). For each individual inferred CF, we compare all edges to the edges of the simulated CFs to determine the true/false positive/negative edge assignments (Supplementary Fig. 2). True positive (TP) edges are edges in a single inferred CF that are also present in a single simulated CF. Thus, in both the inferred and simulated CFs these sequences were grouped together. Similarly, false positive (FP) edges are edges in a single inferred CF that are not found in any of the simulated CFs. True negative (TN) edges are potential connections between sequences that were neither found in the inferred nor simulated CFs. Finally, false negative (FN) edges are edges in an individual simulated CF that are not found in any inferred CF. Since we have many more TN compared to TP cases, the accuracy ((TP + TN) / (TP + TN + FP + FN)) and specificity (TN / (TN + FP)) will always be high and not discriminative between the approaches. Therefore, we report the sensitivity (proportion of TP edges correctly identified by the methods (TP/(TP + FN)), the precision (proportion of TP edges that are actually present in the simulated CFs (TP/(TP + FP)), the F1 score defined as the harmonic mean of precision and sensitivity (2TP/(2TP + FP + FN), and the Jaccard Index, which can be interpreted as a measure of overlap between the ground truth and inferred CFs with a focus on the TP and ignoring the TN (TP / (TP + FN + FP)).

### Effect of approach, sequencing depth and mutation load on outcome measures

The results from CF reconstruction may depend not only on the used approach, but also on dataset characteristics such as mutational load and sequencing depth. To account for the effect of these covariates, we used linear mixed effect regression using the following random intercept model:

$${\boldsymbol{y}}_{\boldsymbol{ij}}={\boldsymbol{\beta}}_{\textbf{0}}+\sum_{\boldsymbol{k}=\textbf{1}}^{\boldsymbol{A}-\textbf{1}}{\boldsymbol{\gamma}}_{\boldsymbol{k}}{\boldsymbol{D}}_{\boldsymbol{ik}}+{\boldsymbol{\beta}}_{\textbf{1}}{\boldsymbol{x}}_{\textbf{1}\boldsymbol{ij}}+{\boldsymbol{\beta}}_{\textbf{2}}{\boldsymbol{x}}_{\textbf{2}\boldsymbol{ij}}+{\boldsymbol{u}}_{\boldsymbol{j}}+{\boldsymbol{e}}_{\boldsymbol{ij}}$$

$${\boldsymbol{e}}_{\boldsymbol{ij}}\sim \boldsymbol{N}\left(\textbf{0},{\boldsymbol{\sigma}}_{\boldsymbol{e}}^{\textbf{2}}\right)$$

$${\boldsymbol{u}}_{\boldsymbol{j}}\sim \boldsymbol{N}\left(\textbf{0},{\boldsymbol{\sigma}}_{\boldsymbol{u}}^{\textbf{2}}\right)$$

where *y*_*ij*_ is the outcome measure (e.g., number of clonal families), *β*_0_ is the overall fixed intercept, *β*_1_, *β*_2_ are the regression coefficients for the explanatory variables *x*_*ij*_ (sequencing depth and mutation load). Approach is a nominal categorical variable represented with *A-1* dummy variables *D*_ik_, with *A* representing the number of approaches we evaluate. D_ik_ = 1 if observation *i* was obtained with approach *k* or 0 otherwise. The model *A-1* regression coefficients *γ*_*k*_ were estimated using approach A3 (absolute threshold (AA)) as the reference (i.e., intercept). *β*_0_ + *u*_*j*_ is the random intercept for the dataset, and *e*_*ij*_ are the residuals. Index *j* denotes the dataset (D1 – D8) and index *i* = 1, … .,n_i_, with n_j_ the number of observations in dataset *j*. Using *lmer* and *lmerTest* [46, 47] R package, we represented this model as

$$\boldsymbol{y}\sim \textbf{1}+\boldsymbol{Approach}+\boldsymbol{Mutation}\ \boldsymbol{load}+\boldsymbol{Sequencing}\ \boldsymbol{depth}+\left(\textbf{1}\ |\ \boldsymbol{Dataset}\right)$$

Thus, the effect of the dataset was modelled as a random intercept. The approach was modelled as a fixed categorical variable. Mutation load and sequencing depth were included as fixed continuous variables. We omitted other terms (e.g., interactions and random slopes) because these could not be fitted due to the limited amount of data. We excluded SCOPer (approach A7 and A8) from all models, since this method deviated too much from the other approaches (see Results section).

We checked the influence of dataset D4 (single cell) on the model results since its sequencing depth and, therefore, the number of CFs was very low (Table 2). This dataset hardly affected the results of the regression and was only removed in one of the models (Table 3). For the regression model of the Gini-Simpson index we removed dataset D3 (RA), which was considered an outlier based on the diagnostic plots we made. We checked the influence of individual outlier observations, which we defined as any data point exceeding q75 + 1.5*IRQ where q75 represents the 75th percentile and IQR the interquartile range (IRQ = q75 – q25). These outliers correspond to the outliers shown in the boxplots. We tested the effect of scaling (mean centering and unit variance) the mutation load and sequencing depth since their scales are very different, and we tested the effect of log-scaling the outcome measures. For all regression models (i.e., with and without outlier removal, scaling, log transformation) we visually checked the model for various assumptions (normality of residuals, normality of random effects, linear relationship, homogeneity of variance, multicollinearity), and the outcome versus the predicted outcome values and, subsequently, selected the most appropriate model. Following model fitting we calculated all pairwise contrasts, using the R package *emmeans* [48], between the approaches using the Holm–Bonferroni [49] method to control the family-wise error rate in the 28 comparisons.

**Table 3:** Results of seven regression models. For each model (outcome measure) we indicate if (i) outliers and/or dataset D3/D4 were removed, (ii) covariates were standardized to zero mean and unit standard deviation, and (iii) outcome measures were log-scaled. For each model the overall significance of the model (ANOVA) and the significant model coefficients are shown. The +/− indicate a positive/negative effect in relative to the model intercept (approach A3). Note that the pairwise comparison for A5-A9 is just above our threshold of 0.05. The four pairwise comparisons shown in bold correspond to the pre-defined comparisons shown in Fig. 2. The asterisk denotes comparisons between CF reconstruction methods (A3-A10). P_adj_ is the Holm–Bonferroni adjusted *p*-value. A = approach, SD = sequence depth; ML = mutation load

Outcome variable	Removal outliers and/or D3/D4	Covariate standar- dization	Log10 scaling	Overall significance (ANOVA)	Significant model coefficients (*p*-value)	Significant pairwise comparisons (p_adj_)
Number of Clonal Families (CF)	Outliers	Yes	Yes	A (*p* < 0.001) SD (*p* < 0.001) ML (*p* < 0.001)	-A6 (< 0.05) -A9 (< 0.05) +SD (< 0.001) -ML (< 0.001)	A2-A6 (0.0024) A2-A9 (0.0011) A10-A6 (0.0229)* A10-A9 (0.0120)*
Singletons	D4	No	Yes	A (*p* < 0.001) SD (*p* < 0.001) ML (*p* < 0.001)	+A2 (< 0.05) -A6 (< 0.05) -A9 (< 0.001) -A10 (< 0.05) +SD (< 0.001) -ML (< 0.001)	A1-A9 (0.0104) **A2-A4 (0.0414)** **A2-A5 (0.0191)** A2-A6 (<.0001) A2-A9 (<.0001) A3-A6 (0.0371)* A3-A9 (0.0037)* A4-A9 (0.0246)* A5-A9 (*0.0521*)* A10-A2 (0.0002)
Mean CF size	None	No	Yes	A (*p* < 0.0001) SD (*p* < 0.05) ML (*p* < 0.001)	+A6 (< 0.05) +A9 (< 0.05) -SD (< 0.05) +ML (< 0.001)	A2-A6 (0.0019) A2-A9 (0.0008) A10-A6 (0.0183)* A10-A9 (0.0094)*
Number of Dominant Clones (0.5%)	None	Yes	Yes	ML (*p* < 0.001)	+ML (< 0.001)	None
D50	Outliers	No	Yes	A (*p* < 0.05) SD (*p* < 0.001) ML (*p* < 0.001)	+SD (< 0.001) -ML (< 0.001)	None
Gini-Simpson index	Outliers D3	Yes	Yes	A (*p* < 0.05) SD (*p* < 0.001) ML (*p* < 0.001)	+SD (< 0.001) -ML (< 0.01)	A2-A6 (0.0463)
Shannon index	Outliers	No	No	SD (*p* < 0.001) ML (*p* < 0.001)	+SD (< 0.001) -ML (< 0.001)	None

### Comparison of the ground truth to the results of different approaches

We compared the ground truth from the simulated data to the results obtained with different approaches for CF reconstruction. We excluded A1 (unique junctions), A2 (subclones), and SCOPer (A7, A8) from these comparisons. We used a repeated-measures linear mixed-effect model to determine the significant differences with the ground truth using the following random intercept model:

$${\boldsymbol{y}}_{\boldsymbol{ij}}={\boldsymbol{\beta}}_{\textbf{0}}+\sum_{\boldsymbol{k}=\textbf{1}}^{\boldsymbol{A}-\textbf{1}}{\boldsymbol{\gamma}}_{\boldsymbol{k}}{\boldsymbol{D}}_{\boldsymbol{ik}}+{\boldsymbol{v}}_{\boldsymbol{j}}+{\boldsymbol{e}}_{\boldsymbol{ij}}$$

$${\boldsymbol{e}}_{\boldsymbol{ij}}\sim \boldsymbol{N}\left(\textbf{0},{\boldsymbol{\sigma}}_{\boldsymbol{e}}^{\textbf{2}}\right)$$

$${\boldsymbol{u}}_{\boldsymbol{j}}\sim \boldsymbol{N}\left(\textbf{0},{\boldsymbol{\sigma}}_{\boldsymbol{u}}^{\textbf{2}}\right)$$

where *y*_*ij*_ is the outcome measure, *β*_0_ is the overall fixed intercept and approached modeled by dummy variables (*D*_ik_). The model *A-1* regression coefficients *γ*_*k*_ were estimated using approach A0 (ground truth) as the reference (i.e., intercept). *β*_0_ + *v*_*j*_ is the random intercept for the sample, and *e*_*ij*_ are the residuals. Index *j* denotes the simulated sample (S28 – S33; Supplementary Table 1**(S3))** and index *i* = 1,….,n_i_, with n_j_ the number of observations in sample *j*. Using *lmer* and *lmerTest* we represented this model as

$$\boldsymbol{y}\sim \textbf{1}+\boldsymbol{Approach}+\left(\textbf{1}|\boldsymbol{Sample}\right)$$

### Light chain and heavy chain concordance analysis

Recently, it was shown that incorporation of the LC does not significantly improve the CF reconstruction process (Zhou and Kleinstein, 2019b). To confirm this finding, we performed a concordance analysis to establish the potential contribution of the LC. Using the two single cell datasets (D4, D5) from healthy donors for the paired chains we evaluated the concordance of the reconstructed HC-based CFs with the CFs obtained when further partitioning these CFs according to V/J gene name(s) of the paired LCs. A CF is considered concordant if all HC within a CFs are paired to the same LCs (i.e., identical V/J gene name). The proportion of concordant CFs is calculated for each approach.

### Identification of shared clonal families

In addition to the outcome measures that we included in the regression analyses we also aimed to determine how these outcomes affect the identification of shared CFs, as an example of a more downstream analysis. For this, we considered the 3 GC samples from dataset D1 that were derived from the same chronic sialadenitis patient and were expected to share a reasonable number of CFs. We also used the peripheral blood samples (D2) from three different RA patients that were expected to share only few CFs. Finally, we used the samples from D3 that comprises a single synovial tissue sample and two synovial fluid samples from the same patient but different joints, which are also expected to share CFs. Consequently, for each dataset we make three pairwise comparisons between the samples within each dataset. To identify shared CFs, we followed the approach of Setliff and co-workers [50], i.e., we merged the three repertoires (samples) from each dataset while keeping the sample annotation of each individual sequence. Subsequently, we reconstructed the CFs from the merged repertoires and counted the number of CFs that include sequences from multiple samples as a measure for the number of shared CFs.

### Software and code availability

We used the *lme4* (version 1.1–31 [46];) and the lmerTest (version 3.1–3 [47];) R packages to fit the linear mixed effect models. The package *emmeans* (version 1.8.2 [48];) was used to calculate pairwise contrasts between the approaches. The *performance* package (version 0.10.0 [51];) was used for visual diagnostics of the linear mixed effect models. We used dplyr (version 1.0.10 [52];) for data transformations and ggplot2 (version 3.4.0 [53];), ggpubr (0.5.0), grid, and RColorBrewer (version 1.1.3 [54];) for visualization. Python 3.9.7 (https://www.python.org) was used for CF inference and further analysis. NCBI IgBlast-1.17.1 [38] was used for V and J genes assignment. Change-O (version 1.2.0 [13]), SCOPer [15], alignment-free method [17], and partis [14] were used for CF inference. Data processing, CF inference pipelines including the code of Change-O, SCOPer and the alignment free CF inference approaches and approach evaluation code is available on GitHub https://github.com/EDS-Bioinformatics-Laboratory/BCRCF.

## Results

### Overall results

**Fig. 3:**
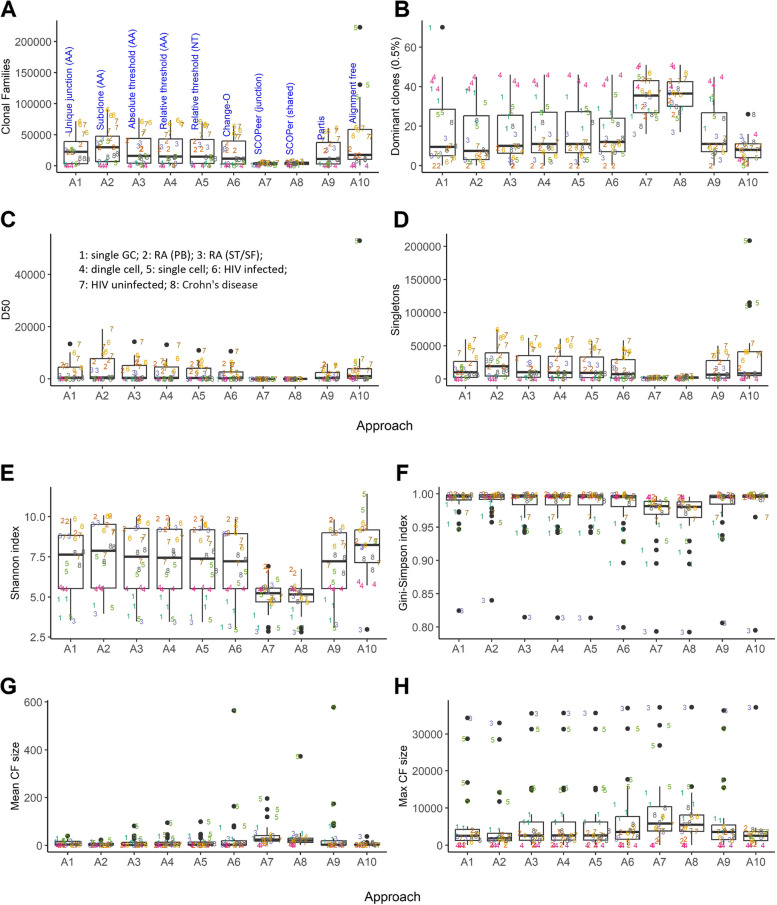
Overall results of the CF reconstruction approaches applied to eight datasets. **(A)** Number of CFs. **(B)** Dominant CFs. **(C)** D50. **(D)** Number of singletons. **(E)** Shannon index. **(F)** Gini-Simpson index. (**G**) Mean CF size. (**H**) Maximum CF size. Numbers in the boxplots correspond to the datasets. The lower and upper hinges correspond to the 25th and 27th percentiles. Black lines show the median. Whiskers denote 5th and 95th percentiles. Each dot represents a sample. Dots beyond the Whiskers represent outliers

We applied each of the 10 approaches to all three samples in our datasets (Table 1). However, A10 (alignment-free method) was not applied to dataset D1 (chronic sialadenitis) due to the absence of multiple individuals in this dataset. Additionally, there were no multiple individuals in the original study, preventing the use of additional data from the original experiment. For the application of approach A10 to D3 for which we also used samples from a single patient, we used an additional sample from another RA patient from the same study as the reference. Fig. 3 shows the overall results of the CF reconstruction for eight outcome measures, i.e., the number of CFs, the number of dominant CFs, D50, the number of singletons, the Shannon and Gini-Simpson diversity indices, and the mean and max CF size. As expected, the differences between the datasets are larger than the differences between the three samples within each dataset.

We observe that approach A1 (unique junction sequences) and A2 (subclones) do not seem to largely deviate from the other approaches despite that A1/A2 do not represent real CFs. SCOPer (A7 and A8) largely deviates from the other approaches and results in far fewer CFs while the number of dominant CFs is inflated resulting in a lower D50. Consequently, also the diversity is much smaller compared to the other approaches. The difference in the other outcome measures is less pronounced for A7/A8. We assumed that the reconstructed CFs by SCOPer are mostly incorrect and, therefore, left out from most of the analyses presented below. This assumption is confirmed by our simulations (see below). The reason for this large deviation is not entirely clear but is likely related to the ‘eigen-gap’ procedure that determines the number of CFs. For dataset D4 (single cell) we find a very low number of CFs and singletons and relatively large number of dominant clones due to the very low sequencing depth. The CFs are also of small size compared to the other datasets. The alignment-free method (A10) results in a very large number of clones for single-cell dataset D5 in contrast to the other approaches. The reason for this is unclear but could be related to the increased number of unique sequences due to its high sequencing depth and, hence, an increased number of k-mers found in these sequences leading to more CFs. More generally, we observe that the mean and maximum CF sizes obtained for D5 are relatively large and mainly a result of the much larger sequencing depth. Also, D3 shows some very large CFs that, however, do no inflate the number of dominant clones compared to other datasets. The Gini-Simpson index, which is dominated by the more frequent CFs, shows similar values (i.e., between 0.8 and 1.0) across all datasets and approaches, but part of the datasets show a relatively low diversity (i.e., D1: single GC; D3: RA ST/SF; D5: single cell; D7: HIV uninfected). A similar observation is made for the Shannon index except for dataset D7, which is now more closely to the median value, indicating that D7 diversity is mainly associated with larger CFs.

In summary, Fig. 3 shows that, overall, all approaches seem to give similar results except for SCOPer (A7, A8). However, the variation in the datasets may, to some extent, obscure the true differences between the approaches. Moreover, without controlling for dataset variability, also approaches A1 (unique junction sequences) and A2 (subclones) do not deviate largely from CFs reconstructed by any of the other methods.

### Sequencing depth and mutation load affect outcome measures

To determine if the 10 approaches lead to differences in the outcome measures, we established if such differences are due to intrinsic differences of the used methodologies or, alternatively, are (partially) caused by dataset characteristics (e.g., sequencing depth, mutational load). Therefore, we fitted a linear random intercept model to account for these covariate contributions. We did not include SCOPer (A7 and A8) in this regression analysis. Table 3 shows the results from the regression analyses. The regression models (ANOVA) show that the chosen approach has an effect on the number of CFs, the number of singletons, the mean CF size, D50, and the Gini-Simpson index but not on the number of dominant clones nor the Shannon index. The sequence depth and/or mutation load have an effect on all outcome measures. From the sign of the significant model coefficients, we see that the number of CFs and singletons increases, as expected, with the sequence depth and decreases with the mutation load relative to the model intercept (Supplementary Fig. 3 and 4). The decrease in the number of CFs and singletons with mutation load can be explained by the assumption that CFs that carry more mutations are more mature (i.e., have gone through more GC cycles) and, consequently, the corresponding samples have fewer but larger CFs, which is confirmed by the positive mutation load sign for the mean CF size and the number of dominant clones. As a result of the increased size of the CFs, the repertoires will also contain fewer singletons since the larger CFs have more chance of being sequenced at a certain depth at the expense of these singletons. As a consequence, we also see that the mutation load has a negative effect on D50 and both diversity indices. In summary, we find that after accounting for the variability in the datasets, in particular the sequencing depth and mutation load, the reconstruction approach has an effect on part of the outcome measures including the number of CFs.

### The approach for CF reconstruction has a limited effect on the outcome measures

The regression model established that the approach has an effect on part of the outcome measures and, therefore, we subsequently inspected the regression coefficients (γ) for the approaches to identify the specific approaches responsible for this. From Table 3 and Supplementary Fig. 5 we see that only a few approaches (A2, A6, A9, and A10) positively or negatively affect the number of CFs, singletons, mean CF size, D50, and the Gini-Simpson index, compared to A3 (absolute threshold (AA)) that was used as the reference in the linear model. For example, approach A6 (Change-O) gives fewer CFs and singletons compared to approach A3. Note that for D50 the ANOVA results in a significant overall effect for approach, while none of the individual model coefficients were significant, which indicates a correlation between the approaches. Based on the model we additionally determined all significant pairwise comparisons between the approaches. Since in these comparisons we control for the family-wise error rate using the Holm-Bonferroni method, we do not find a significant difference for A3-A6 and A3-A9 whose initial uncorrected *p*-values (*p* = 0.0168 and *p* = 0.0284 respectively) now fall below our threshold of p_adj_ = 0.05. Only four of the predefined comparisons shown in Fig. 2 turned out to be significant. These are the differential number of singletons between A2 (subclones) and A4 (relative threshold (AA)), and A2 and A5 (relative threshold (NT)), and the differential number of CFs and mean CF size between A9 (partis) and A10 (alignment free).

We find a mean number of singletons of 28,028, 21,275, and 20,407 for A2, A4 and A5 respectively. Thus, as expected, clustering subclones (A2) into CFs (A4, A5) results in fewer singletons. Although the comparison between A2 and A3 (absolute threshold (AA)) is not statistically significant, A3 results in a similar number of singletons (22,347) compared to A4/A5. Interestingly, there is no significant difference in the number of CFs between A2 and A3/A4/A5 (36,756, 30,409, 29,336, and 28,513 respectively). Note that for these comparisons the uncorrected *p*-values for A2-A3, A2-A4, and A2-A5 are *p* = 0.0734, *p* = 0.0372, and *p* = 0.0257 respectively. For the A9 to A10 comparison we determine that these result in an average of 23,893 and 43,217 CFs respectively with mean sizes of 40,593 and 5612 sequences. Thus, A10 results in many more CFs but of much smaller size (Supplementary Table 1 (S3)). These differences likely stem from the fact that these methods utilize the full sequence differently, and because partis (A9) performs a VJ-partitioning in contrast to the alignment free approach (A10). Based on these regression results we cannot conclude that there is a difference between using a nucleotide or amino acid representation (A4 vs A5), nor that there is a difference between using a relative, absolute, or sample based threshold (A3 vs A4 and A5 vs A6).

Apart from these, a priori defined comparisons of interest (Fig. 2) there are several other significant pairwise comparisons resulting in differences for the number of CFs, singletons, mean CF size or Gini-Simpson index. In contrast, the number of dominant clones, D50 and the Shannon index are not affected by the approach. The significant difference in the number of CFs and mean CF size when comparing A6 (Change-O) and A10 (alignment free) can be due to the VJ-partitioning in A6, the use of the full sequence in A10, or the use of different thresholds. However, this could also be caused by the inflated number of CFs produced by A10 which seems to be incorrect (see simulations below). There doesn’t seem to be any difference between approaches (A3, A4, A5, A6) that use VJ-partitioning and junction similarity for any of the outcome measures except for A3 (absolute threshold (AA)-A6 (sample-based threshold (NT)). There are several approaches resulting in differences for the number of singletons but these will be generally of less interest and also don’t seem to affect the diversity of the repertoires. We also note that there is no difference between A1 (unique junctions) and A2 (subclones), showing that VJ-partitioning is not required per se to get an indication about the number of subclones. In addition, A2 (subclones) gives a different number of CFs compared to all other methods except A10 (alignment free) that seems to produce a too large number of CFs. It also seems fair to conclude that using the full sequence (A9, A10) in contrast to only using the junction sequence does not have a large effect on the number of CFs. We could not make any conclusion about the advantage of using shared mutations (A7/A8, SCOPer) since this approach was not included in the regression model.

In summary, comparing the approaches that reconstruct CFs (A3-A10), we observe that only eight comparisons are significant for three of the outcome measures (number of CFs, singletons, and mean CF size). Four of these comparisons affect the number of singletons. Moreover, four of these comparisons involve A10 (alignment free) that seems to produce a too large number of CFs. For this reason, we conclude that the specific approach for CF reconstruction only has a limited effect on the outcome measures that we tested. A simple method for CF reconstruction like A4 (setting a relative similarity threshold for the amino acid junction sequence) does perform equally well as more sophisticated methods like A9 (partis).

### Deviation from ground truth varies between the approaches

The results from our regression model and the pairwise comparisons reveal differences between the approaches but does not inform us which approach is best since the ground truth for any of the included datasets is unknown. Therefore, we also simulated CFs and applied the approaches to the simulated data to establish the performance of the approaches (Supplementary Table 1 (S4)). For this evaluation we included A7/A8 (SCOPer). As expected, the number of TNs, representing pairs of sequences that are absent in both the simulated and inferred CFs, is two orders of magnitudes larger compared to the other number of cases (TP, FP, FN; Supplementary Fig. 6). The differences between the samples are only partially caused by the differences in the number of sequences in each simulated sample. Normalizing the total number of cases with respect to the largest number of cases (dataset D11, sample 1) still shows differences between the samples (Supplementary Fig. 7). In addition, we see that a larger number of sequences does not automatically lead to more TP cases given that dataset D11/sample 1 has more sequences compared to the other samples but has the fewest number of TP cases. The reason for this may lie in the different structure of the simulated dataset D11, which contains a larger number of CFs but on average they are smaller in size compared to CFs from datasets D10 and D12.

Approaches A1 (unique junctions) and A2 (V-junction-J) represent unique sequences and, therefore, the number of cases (i.e., TPs, TNs, FPs, FNs) cannot directly be compared to the number of cases observed for the other eight approaches because there is no relation between the number of CFs and the number of unique junctions/subclones they may include. We observe a low number of FP cases for A1 and A2 corresponding to identical sequences that were part of different simulated CFs but grouped together after CF inference. The probability that identical junction sequences occur in different simulated CFs is very low but may also occur in real CFs. Similarly, the probability for identical subclones to occur in different simulated/real CFs is low. Therefore, we did not aim to account for this in our simulation approach. More interestingly is the comparison between approaches A3 to A10. The number of TP and TN cases are similar for all approaches including SCOPer (A8 and A9) although the number of TP for A10 (alignment free) is somewhat lower and shows more variability. However, the number of FPs and FNs produced by SCOPer is very different from the other approaches. It seems that SCOPer erroneously groups sequences into single CFs (resulting in far fewer CFs (Fig. 3(A)) and, at the same time but less commonly compared to the other approaches, separates sequences from a single CF into different CFs. In general, we observe that differences between the approaches are mainly caused by the number of FPs they produce. The approaches A4-A6, A9, and A10 result in a different number of TP cases (*p* = 0.002 and *p* = 0.001 for the unformalized and normalized number of cases; Kruskal-Wallis rank sum test) and also the subset A3, A4, A5, and A6, which use VJ-partitioning but different junction-based similarity thresholds affect the number of FP cases (*p* = 0.004 and *p* = 0.0004). No significant differences occur in the number of TNs/TPs/FNs for these two groups.

**Fig. 4:**
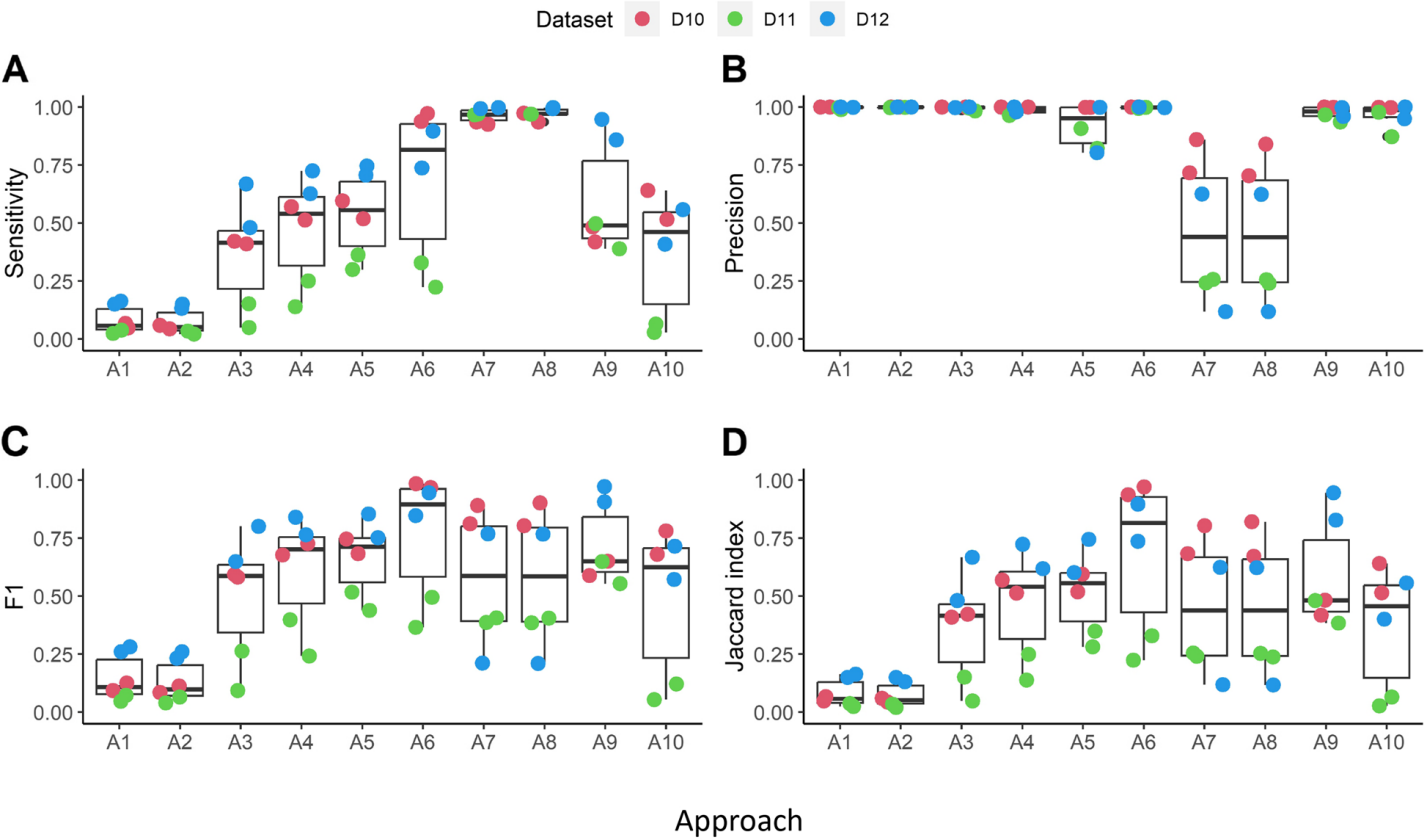
Performance as measured by the sensitivity, Precision, F1 and Jaccard index for all approaches applied to two samples from 3 datasets. The lower and upper hinges correspond to the 25th and 27th percentiles. Black lines show the median. Whiskers denote 5th and 95th percentiles. Each dot represents a sample. Dots beyond the Whiskers represent outliers

In Fig. 4 we report the sensitivity, precision, F1, and Jaccard index as overall performance measures for the 10 approaches we evaluated. As expected, approach A1 (unique junction) and A2 (subclones) show a very poor performance since these do not represent CFs. Nevertheless, A1/A2 produced a similar number of CFs compared to the other approaches (Fig. 3**,** Table 3) but with fewer FP and more FN cases. The precision for all approaches except SCOPer (A7, A8) is very high and, therefore, also not discriminative between the methods. In general, the number of FPs is very low compared to the number of TPs, resulting in a high precision indicating that the sequences that are grouped together indeed make part of the same clone. The sensitivity shows larger differences between the approaches indicating that these methods perform differently with respect to correctly clustering pairs of sequences into CFs. A6 (Change-O) performs the best in this regard, while A3, A9 and A10 only show a sensitivity of about 0.5 showing that the probability of correctly grouping pairs of sequences, given that this pair is truly part of the same CF, is only about 50%. That is, about 50% of these pairs are not grouped together (FNs). In addition, more complex approaches like partis (A9) and the alignment free method (A10) do not result in an increased sensitivity. The precision of all approaches is very high (except for SCOPer; A7, A8) and a result of the low number of FP occurrence of sequence pairs within reconstructed CFs. The F1 score, which can be interpreted as a balance between sensitivity and precision ability to both capture positive cases (recall) and be accurate with the cases it does capture (precision). The F1 score will be low if the sensitivity and precision are very different, or if one of these performance measures has a low value. Fig. 4 shows that most approaches have a similar F1 score although A6 (Change-O) seems to do better in identifying the TP cases correctly (high sensitivity and precision). Note, however, that the precision of all approaches is very high and, therefore, the F1 score has a similar trend as the sensitivity. Finally, the Jaccard index shows the overlap between the ground truth and the number of inferred sequence co-occurrences in the inferred CFs with a focus on the TP and ignoring the TN. The trend is similar to the sensitivity and F1 score.

**Fig. 5:**
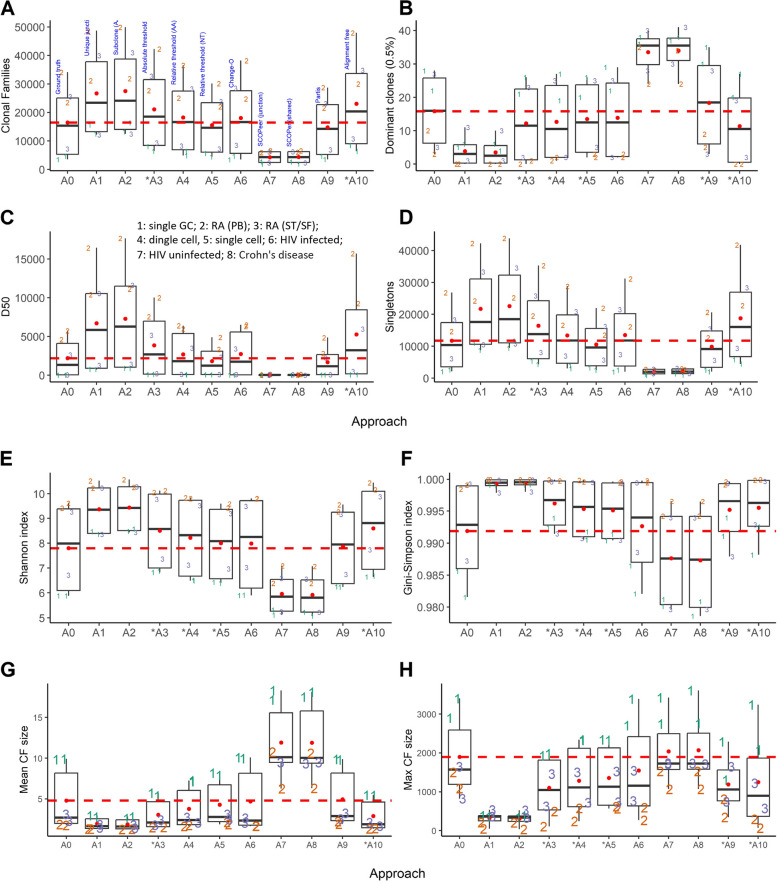
Comparison of outcome measures to ground truth. Approach A0 represents the known ground truth of the simulated data. The red dashed lines show the mean ground truth. Red dots represent mean values. Asterisks on the x-axis represent significant differences with the ground truth based on the repeated-measures model (p < 0.05). A1, A2, A7, A8 were excluded from the model. The lower and upper hinges correspond to the 25th and 27th percentiles. Black lines show the median. Whiskers denote 5th and 95th percentiles. Each dot represents a sample. Dots beyond the Whiskers represent outliers

Figure 5 shows the outcome measures for all approaches applied to the simulated data in comparison to the ground truth (A0). Again, approaches A1, A2 and SCOPer (A7, A8) deviate largely from A0 although the maximum CF size produced by SCOPer very close to the ground truth, but a direct result of too large number of FPs. Approach A10 significantly (*p* < 0.05) deviates from the ground truth for all outcome measures, while approach A3 (Rel. Threshold (AA)) significantly deviates (*p* < 0.05) from the ground truth for all outcome measures except D50. Approach A6 (Change-O) is the only approach that always agrees with the ground truth.

In summary, our simulations show the A1 (unique junctions) and A2 (subclones) should not be used as surrogates for CFs. In addition, more sophisticated methods like partis and the alignment free method do not outperform more simplistic methods. Finally, Change-O (A6) does seem to perform a little better compared to the other methods and is always in agreement with the ground truth. At the same time, we see there is room for improvement and that performance may differ for different datasets.

### Approaches differ in their ability to reconstruct CFs when not considering the LC

**Fig. 6:**
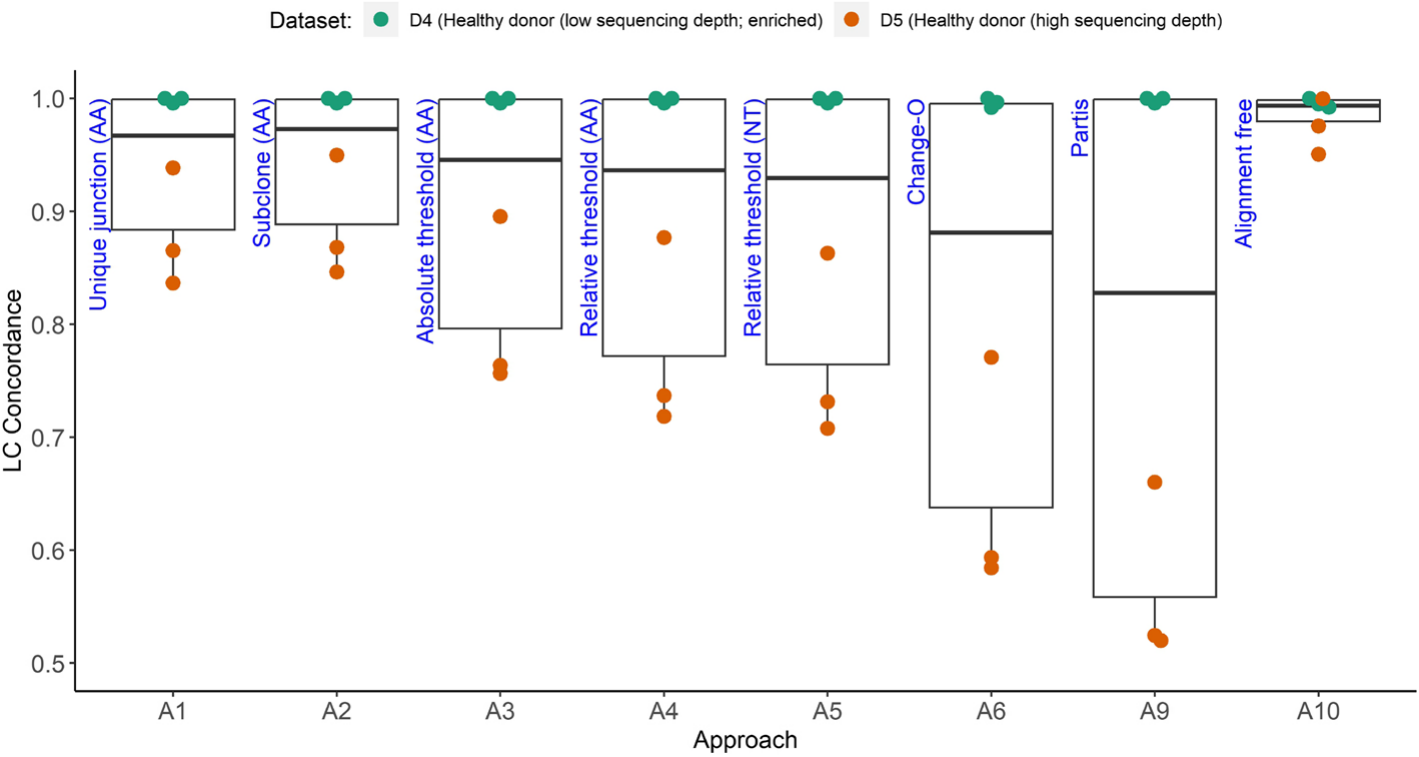
Heavy and light chain concordance. Concordance was calculated for all approaches except SCOPer (A7, A8) using the three samples from the single cell datasets D4 (low sequencing depth) and D5 (high sequencing depth). Concordance was defined as the fraction of CFs that are split in two or more CFs if the LC would be incorporated in the reconstruction process

To investigate the potential influence of LCs on the CF reconstruction we performed a concordance analysis to determine the potential of the LC to split CFs if it is incorporated in the reconstruction process (Fig. 6**;** Supplementary Table 1 (S5)). For D4 (healthy donor; low sequencing depth) we observe a very high concordance (approximately 1) for all approaches, indicating that the LC has virtually no effect on the reconstruction. For dataset D5 (healthy donor, high sequencing depth) we observe a larger range of concordance (0.5–0.95) indicating that the LC may affect the reconstruction of CFs. In particular, we observe that for A6 (Change-O), which came closest to the ground truth (Fig. 5) and A9 (partis) the effect of the LC might not be neglected. The concordance we find for A6 is lower compared to previous research in which a concordance of over 80% was established for HC-based CFs when reconstructed with SCOPer or Change-O [18]. In another study that used partis, it was claimed that for larger samples the concordance could even become much lower [19]. Approach A10 (alignment free) results in a high concordance for D5, which is a result of the many smaller but incorrect CFs produced by this method (Figs. 3 and 4). The high concordance of D4 is a result of its low sequencing depth and because the D4 repertoires were generated from sorted SARS-CoV-2 spike-reactive B cells from unexposed and unvaccinated healthy donors resulting in an enrichment of specific HCs and LCs [42] (Supplementary Table 1 (S5)).

In summary, we conclude that different approaches differ in their ability to correctly reconstruct CFs when not considering the LC. Moreover, Change-O (A6) does not outperform the other approaches despite our simulations showing that A6 closely resembled the ground truth for all outcome measures.

### Identification of shared clonal families

**Fig. 7:**
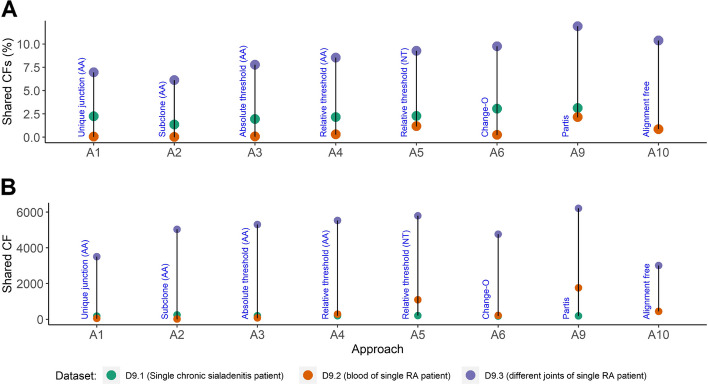
**A** Percentage and **(B)** absolute number of shared CFs. We identified the number of shared clones among three samples identified by different approaches (except SCOPer). D9.1: three single GC samples from a single chronic sialadenitis patient. D9.2: three samples from peripheral blood from three RA patients. D9.3: three Synovial tissue/fluid samples from a single RA patient but different joints. Note that A10 (alignment free) could not be applied to dataset D9.1

In addition to the outcome measures we included for the regression analysis, we also asked if different approaches result in different numbers of identified shared CFs between samples of three selected datasets. We excluded SCOPer (A7, A8) from this analysis. Overall, the number of shared CFs ranges between 0.3 and 12% (Fig. 7**;** Supplementary Table 1 (S6)). For D2 with samples from three patients we find 0.02–2.2% shared CFs. This is lower than found in a previous study with about 0.02% shared CFs among 10 subjects sequenced at a much higher depth [8]. As expected, the number of shared CFs between patients (D9.2) is lower compared to the shared CFs found in a single patient (D9.1 and D9.2). Within each dataset, the number of shared unique junction sequences (A1) and number of shared subclones (A2) is smaller compared to the number of shared CFs (A3 – A10) except for A1 for dataset D9.1. For dataset 9.2 (single RA patient) we compared the number of shared dominant CFs of the three samples that were obtained from the synovial tissue (ST), synovial fluid (SF) of the left knee, and synovial fluid of the right knee. Two shared dominant CFs were identified by all approaches between the two SF samples. In addition, two and five shared dominant CFs were identified by approach A6 (Change-O) between the ST and SF samples. These numbers of shared dominant clones are in agreement with the numbers reported in an earlier study [27].

In summary, the number of shared (dominant) CFs identified varies between the approaches but is in line with previous research. However, from the limited data we used and without knowing the ground truth, we cannot establish if differences between the approaches are statistically or biologically significant.

## Discussion

In this work, we compared eight different approaches for the reconstruction of CFs in addition to a method that only considers unique junction sequences and a method that only considers subclones. We applied these approaches to different bulk and single cell datasets and simulated data to establish the effect on different outcome measures, the identification of shared clones, and LC concordance.

We showed that most approaches for CF reconstruction perform similarly except for SCOPer (A7, A8) that produces fewer and larger CFs compared to the other approaches, which is likely caused by the similarity measure (eigen-gap) of this method, which was only tested on simulated data (personal communication) [15, 16]. Moreover, the outcomes from approaches A1 (unique junction sequences) and A2 (subclones) deviate much less than expected from other reconstruction approaches, even after controlling for the differences in sequencing depth and mutation load of the various datasets. The alignment free method (A10) seems to incorrectly inflate the number of CFs and resulted in a very large number of CFs for single cell dataset D5. Therefore, based on the experimental data it seems fair to conclude that simple approaches such as only setting a relative similarity threshold for the amino acid junction sequence (A4) perform equally well as more sophisticated methods like A9 (partis).

Our regression analyses did show that sequencing depth and mutation load can have a significant effect on the outcome measures. Increased sequencing depth increases the number of (dominant) CFs and repertoire diversity but decreases the average CF size. Increased mutation load tends to correlate with an increased number of CFs and a decreased CF size, which might be related to the observation that more mature repertoires generally harbors more somatic mutation. Including results from our simulations, it is clear that A1 (unique junctions) and A2 (subclones) should not be used as surrogates for CFs despite their seemingly similarity to other approaches when applied to experimental data. Moreover, the simulations show that more sophisticated methods like partis (A9) and the alignment free method (A1) do not outperform more simplistic methods. Finally, Change-O (A6) seems to perform little better compared to the other methods and is always in agreement with the ground truth. However, Change-O does not outperform the other approaches in the LC concordance analysis. In general, our LC concordance analysis shows that the extent to which the LC will improve the reconstruction of CFs depends on the dataset and the approach. Finally, we showed that the number of shared (dominant) CFs identified varies between the approaches.

We define shared CFs as groups of BCRs from different individuals or different tissues that have BCRs with highly similar or identical sequences. Their existence can be explained by the possibility of Ag-driven CF convergence [55]. Shared CFs are generally identified based on their similarity (which can be defined in several ways). Alternatively, one can attempt to experimentally identify shared CFs using a binding assay if the Ag is known. Since methods for CF reconstruction are applied to single samples, they can never group ‘shared’ CFs together since, by definition, these exist in separate samples. Therefore, in our approach, we first merged different samples together prior to the identification of shared clones. We did not change the similarity thresholds for the identification of shared CFs. However, it is conceivable that for the identification of shared CFs these should be less stringent compared to the thresholds used for CF reconstruction. This would require to have a set of shared CFs that have been verified to bind to the same Ag. Another complication in the identification of shared CFs is the fact that these clones may use different V and J genes. Consequently, any CF reconstruction method that first partitions the sequences based on gene usage will miss large part of the shared CFs. In our approach we also neglected this complication and applied the reconstruction methods as designed. Given the limited amount of data we cannot establish if differences between the approaches are statistically or biologically significant. In general, our results show that there is room to further improve methods for CF reconstruction.

We note that there are limitations to our study. Firstly, we included a limited set of eight datasets (24 samples) which may not be fully representative. Moreover, additional datasets and/or samples would give more power to detect differences between the approaches and/or to include additional terms in our regression model to improve the fit and interpretation. In addition, additional datasets would be required to, for example, determine differences between DNA and RNA-based repertoires, or to determine specific differences for more homogeneous repertoires (like our single GC data) and more heterogeneous repertoires obtained from blood. But also, the inclusion of additional single cell datasets that are less biased in terms of sequencing depth. Secondly, the six simulations we performed restricts the power to detect differences but, nevertheless, give a first indication of the true performance of the different approaches. However, it would be interesting to more systematically simulate data at different sequencing depth, mutation loads, repertoire diversity, distribution of (shared) mutations, etc. Simulations would also enable the identification of effect of sequencing errors (or allelic variants) on CF inference. They affect the similarity between sequences. Because we know CF ground truth in simulations, we can evaluate CF inference errors caused by sequencing errors. Thirdly, in our evaluation we used default settings for approaches A6 – A10, and only one similarity threshold choice for approaches A3-A5. Ideally, all approaches should have been tried at various settings of their parameters to reveal further variability in the results. In addition, we could have included other approaches such as reconstruction that based on all CDR regions, or to use dedicated approaches for reconstruction based on single cell data and that incorporate both the HC and LC [56]. However, since this would significantly increase the amount of computation, we decided not to do this. Moreover, in practice it would also be difficult to decide on the best parameterization without having knowledge about the ground truth or other information to guide the settings. The inclusion of the D gene might further improve CF reconstruction. Several approaches have been proposed for the identification of the D-gene reconstruction [57, 58]. IgBLAST [38] and IMGT/V-QUEST [59] also provide information about sequence D-gene assignments. However, since the D genes are small and variable, the assignment is not always reliable and can provide a source of error. Fourthly, we defined the mutation load based on the V and J genes only since it is currently impossible to reliably determine the mutations in the junction region. Hence, our mutation load underestimates the true number of mutations and, consequently, the effect of mutations may even be larger than currently established by our regression model. Finally, our analyses mainly focused on several basis outcome measures such as number of (dominant) CFs, size, and D50. However, the real interest is to determine if different approaches, and hence differences in these outcome measures, would lead to a different interpretation of the data and a different biological conclusion given the research question.

Currently, a range of approaches to infer CFs from AIRR-seq data have been developed and were published only after we did evaluation [19, 60–62]. In [62] authors proposed to combine probabilistic models that capture the receptor generation and selection statistics with adapted clustering methods to achieve consistently high inference accuracy, their approach automatically leverages the phylogenetic signal of shared mutations in difficult repertoire subsets. In [60] authors proposed an approach based on multi-objective clustering. Their CF inference approach requires V(D)J annotations. Note, that this method uses normalized Levenshtein distance for sequence distance calculation, thus, it is useful for reconstruction of CFs with possible indels due to somatic hypermutation. The essential direction for development of the methodology for CF inference is creation of approaches that use paired HC/LC chain data [19, 61]. This became possible as a result of the development of single cell sequencing technologies (e.g., 10X Genomics, [63]). As a consequence, future BCR CF inference evaluation studies may be performed on larger data with BCR HC/LC paired information.

### Supplementary Information

**Additional file 1: Supplementary Figure 1. **Data simulation pipeline. Simulation approach is an integration of ImmuneSim, Alakazam and SHazaM tools and equally use the data of CF groupings obtained from each of the 10 CF inference approaches. **Supplementary Figure 2. **Determination of the number of TP, TN, FP, and FN. Three simulated CFs (2 singletons) and two inferred CFs are shown.** Supplementary Figure 3. **Overall correlation between the log10(number of CFs) and the standardized sequence depth for all combinations of approach (except SCOPer; A7, A8) and dataset.** Supplementary Figure 4. **Overall trend between the log10(number of CFs) and the standardized mutation load for all combinations of approach (except SCOPer; A7, A8) and dataset.** Supplementary Figure 5. **Summary of significant pairwise comparisons between Approaches.** Supplementary Figure 6. **Number of TP, TN, FP, and FN cases produced by the ten approaches when applied to six samples from three simulated datasets (D10, D11, D12).** Supplementary Figure 7. **Normalized number of TP, TN, FP, and FN cases produced by the ten approaches when applied to six samples from three simulated datasets (D10, D11, D12).

**Additional file 2. **Evaluated  approaches for clonal famility reconstruction. BcR repertoire datasets used to evaluate clonal familty reconstruction approaches. Results of outcome measures for all approaches applied to all datasets. Performance of 10 approaches applied to two samples from three simulated datasets. Heavy chain (HC) and Light chains (LC) in single cell datasets D4 (Claireaux et al, 2022) and D5 (DeKosky et al., 2013). Concordance analysis of HC-based clonal families. Shared Clonal Families. Dominant shared Clonal Families for D9.2 (single RA patient).

## Data Availability

Publicly available datasets were analyzed in this study. This data can be found here: D1: processed sequencing data were deposited on the VDJ server under UUID 8899006209436478995-242 ac118–0001-012, publicly accessible at https://vdjserver.org/community/8899006209436478995-242ac118-0001-012. D2 and D3: the datasets can be found in online repositories. The names of the repository/repositories and accession number(s) can be found below: NCBI, accession ID: PRJNA822925. D4: source data are provided as a Source Data File accompanying https://www.nature.com/articles/s41467-022-32232-0. D5: sequence data, SRA: SRA061316. D6 and D7: data from the HIV cohort can be found in the BioProject PRJNA486667. Data from CMV-seropositive and seronegative healthy controls are deposited as BioProject PRJNA491287. D8: Sequencing data are available from the EGA (EGAD00001005431 – EGAN00001806418EGAN00001806419, EGAN00001806420).
