# Supplementary material for: Systematic evaluation of B-cell clonal family inference approaches
Source: BMC Immunol. 2024 Feb 8;25:13. doi: 10.1186/s12865-024-00600-8 (PMC11370117; doi:10.1186/s12865-024-00600-8)
Supplement: Supplementary file 1 — Additional file 1: Supplementary Figure 1. Data simulation pipeline. Simulation approach is an integration of ImmuneSim, Alakazam and SHazaM tools and equally use the data of CF groupings obtained from each of the 10 CF inference approaches. Supplementary Figure 2. Determination of the number of TP, TN, FP, and FN. Three simulated CFs (2 singletons) and two inferred CFs are shown. Supplementary Figure 3. Overall correlation between the log10(number of CFs) and the standardized sequence depth for all combinations of approach (except SCOPer; A7, A8) and dataset. Supplementary Figure 4. Overall trend between the log10(number of CFs) and the standardized mutation load for all combinations of approach (except SCOPer; A7, A8) and dataset. Supplementary Figure 5. Summary of significant pairwise comparisons between Approaches. Supplementary Figure 6. Number of TP, TN, FP, and FN cases produced by the ten approaches when applied to six samples from three simulated datasets (D10, D11, D12). Supplementary Figure 7. Normalized number of TP, TN, FP, and FN cases produced by the ten approaches when applied to six samples from three simulated datasets (D10, D11, D12). [file 12865_2024_600_MOESM1_ESM.pdf]

Supplementary Material

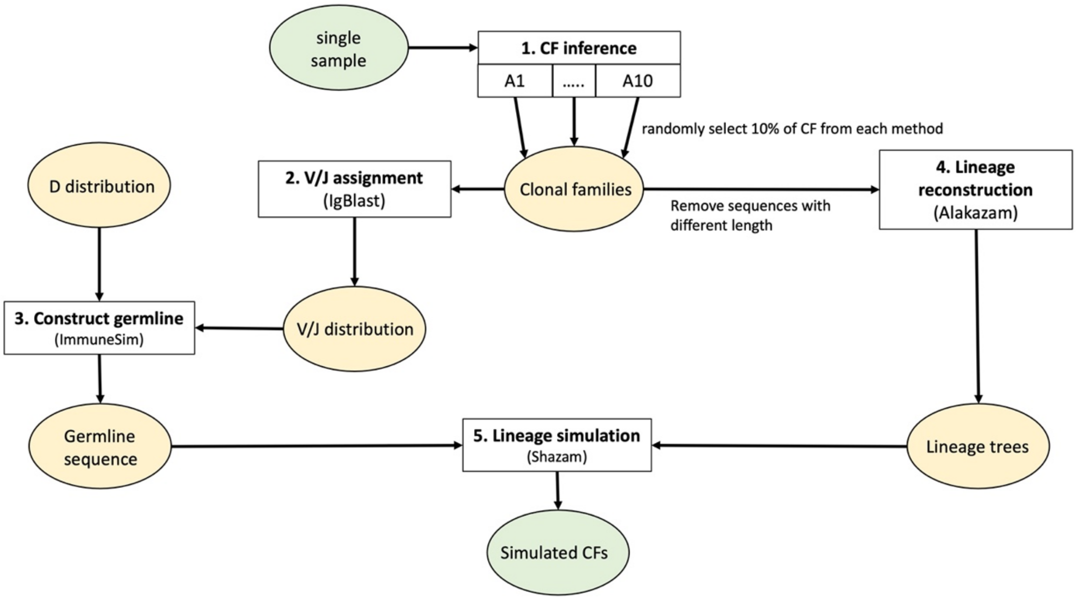

Supplementary Figure 1. Data simulation pipeline. Simulation approach is an integration of ImmuneSim, Alakazam and SHazaM tools and equally use the data of CF groupings obtained from each of the 10 CF inference approaches.

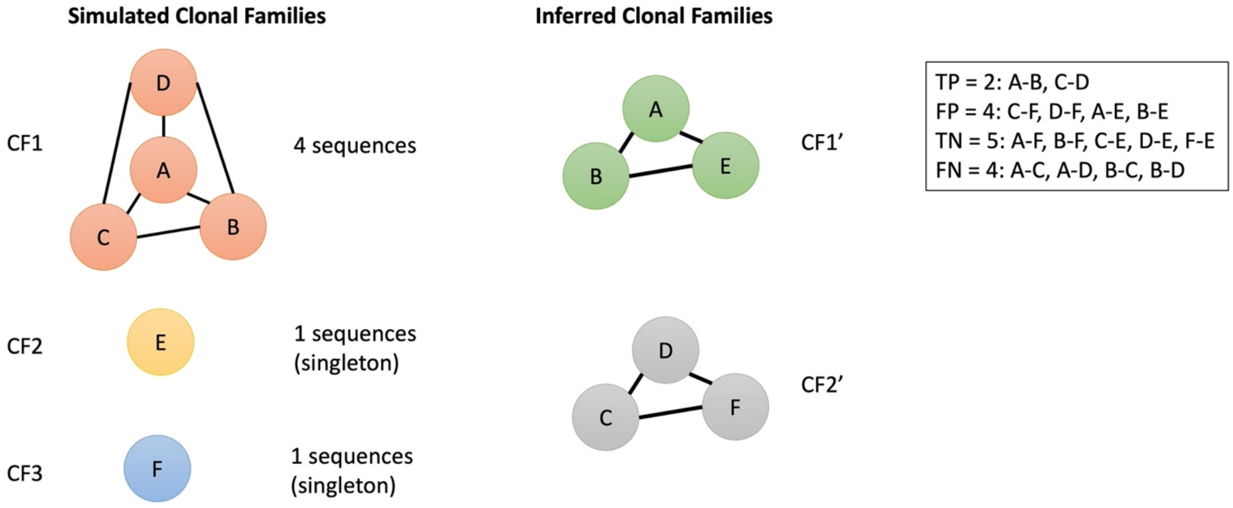

Supplementary Figure 2. Determination of the number of TP, TN, FP, and FN. Three simulated CFs (2 singletons) and two inferred CFs are shown. The letters correspond to unique sequences. We do not take into account the frequency of individual sequences within a clone. All sequences used for the simulated CFs are also assigned to interred CFs. The black lines show connected sequences having a similarity above the threshold as defined by the individual approaches. The counting is done based on the co-occurrence of sequence pairs within a simulated and inferred CF. For example, A-B is a TP since these two sequences occur in the simulated and inferred CF. Sequences C-D occur together in CF2’ and therefore are counted as a TP despite the fact that sequence A and B occur in a different inferred CF. The occurrence of C-F in CF2’ is considered a FP because these two sequences are not part of the same simulated CF. A-F is a TN because these two sequences do not occur together in the simulated nor in the inferred CFs. Finally, A-C is an example of a FN because A-C occur together in a simulated but not in an inferred CF.

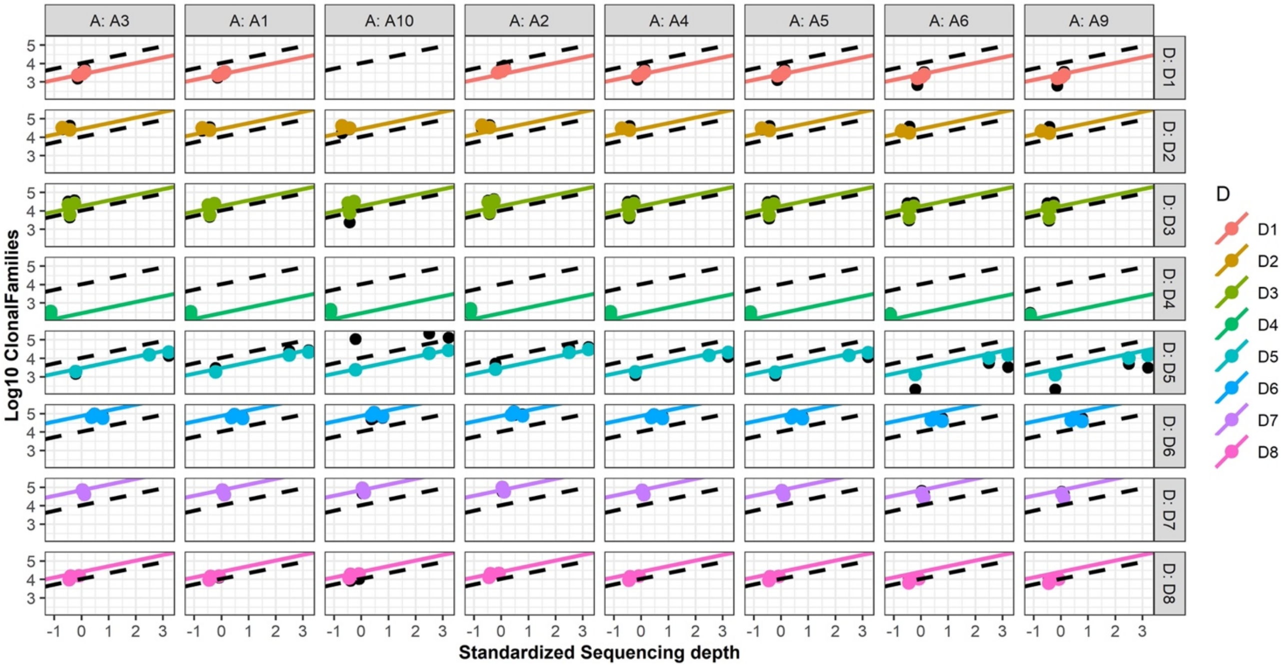

Supplementary Figure 3. Overall correlation between the log10(number of CFs) and the standardized sequence depth for all combinations of approach (except SCOPer; A7, A8) and dataset. The black line corresponds to the fixed intercept β_0 (at the standardized sequence depth of zero). The colored line corresponds to the random intercept $\beta_0 + u_j$. The slope of both lines is determined by the effect of the sequencing depth (β_1). Black points correspond to the actual number of CFs, while the colored points correspond to the number of CFs predicted by the mixed effect model. Note that approach A10 (alignment free) could not be applied to dataset D1 (see main text).

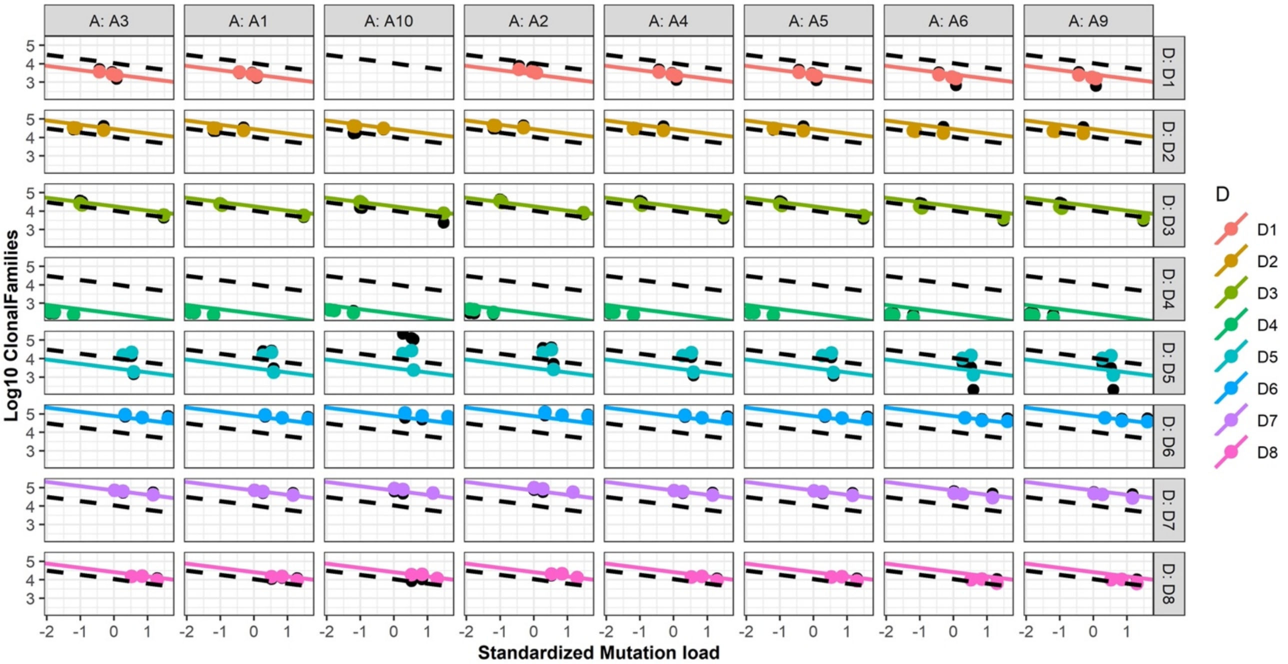

Supplementary Figure 4. Overall trend between the log10(number of CFs) and the standardized mutation load for all combinations of approach (except SCOPer; A7, A8) and dataset. The black line corresponds to the fixed intercept β_0 (at a standardized mutation load of zero). The colored line corresponds to the random intercept $\beta_0 + u_j$. The slope of both lines is determined by the effect of the mutation load (β_2). Black points correspond to the actual number of CFs, while the colored points correspond to the number of CFs predicted by the mixed effect model. Note that approach A10 (alignment free) could not be applied to dataset D1 (see main text).

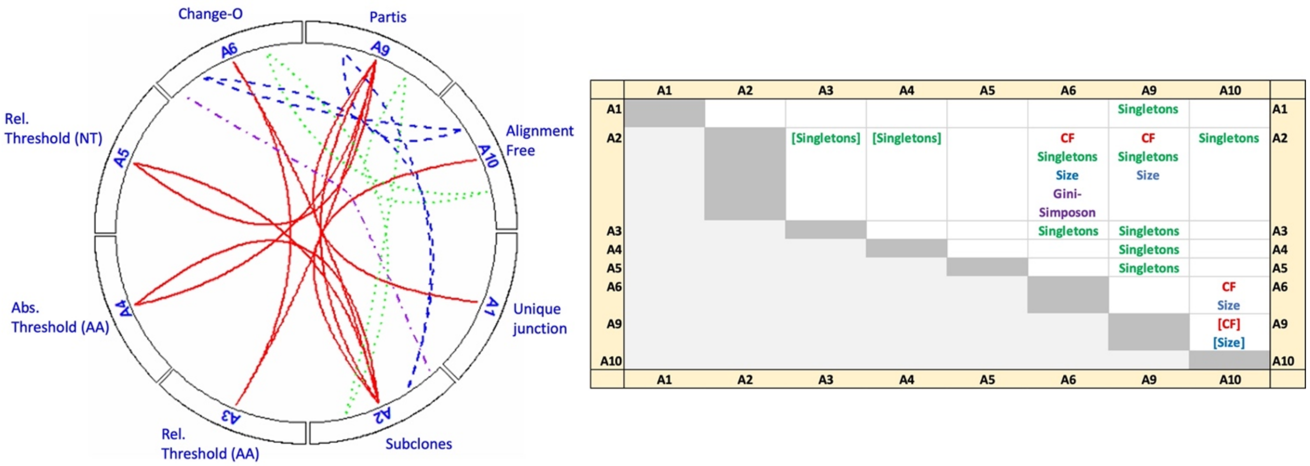

Supplementary Figure 5. Summary of significant pairwise comparisons between Approaches. Blue: CFs; Red: singletons; Green: mean CF size; Purple: Gini-Simpson index. A7 and A8 (SCOPer) were excluded from the comparisons. Outcome measures between brackets denote the initial comparisons of interest as indicated in Figure 2.

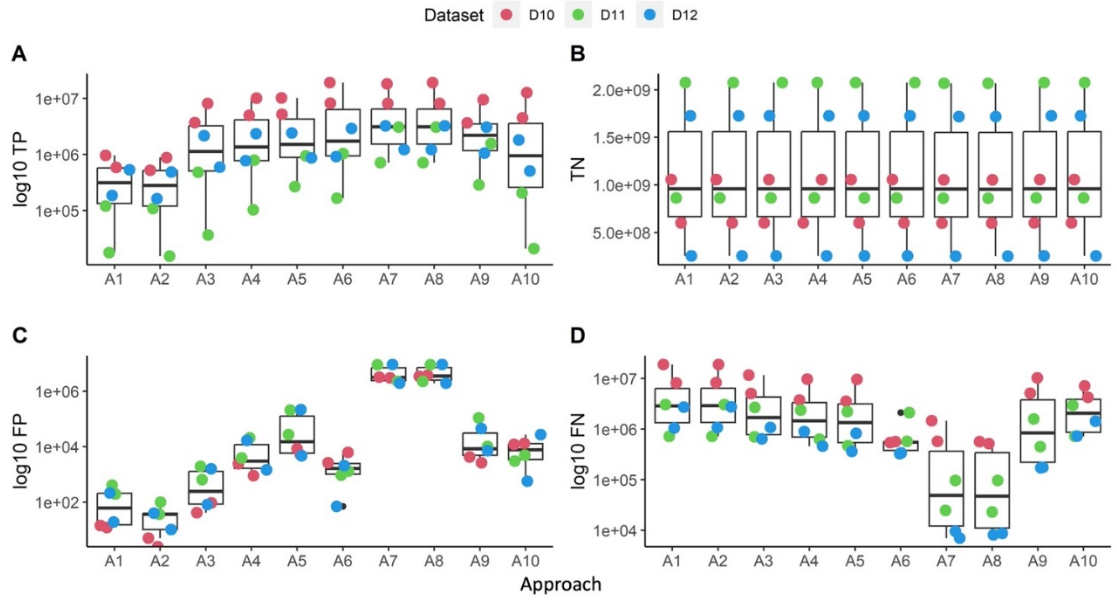

Supplementary Figure 6. Number of TP, TN, FP, and FN cases produced by the ten approaches when applied to six samples from three simulated datasets (D10, D11, D12).

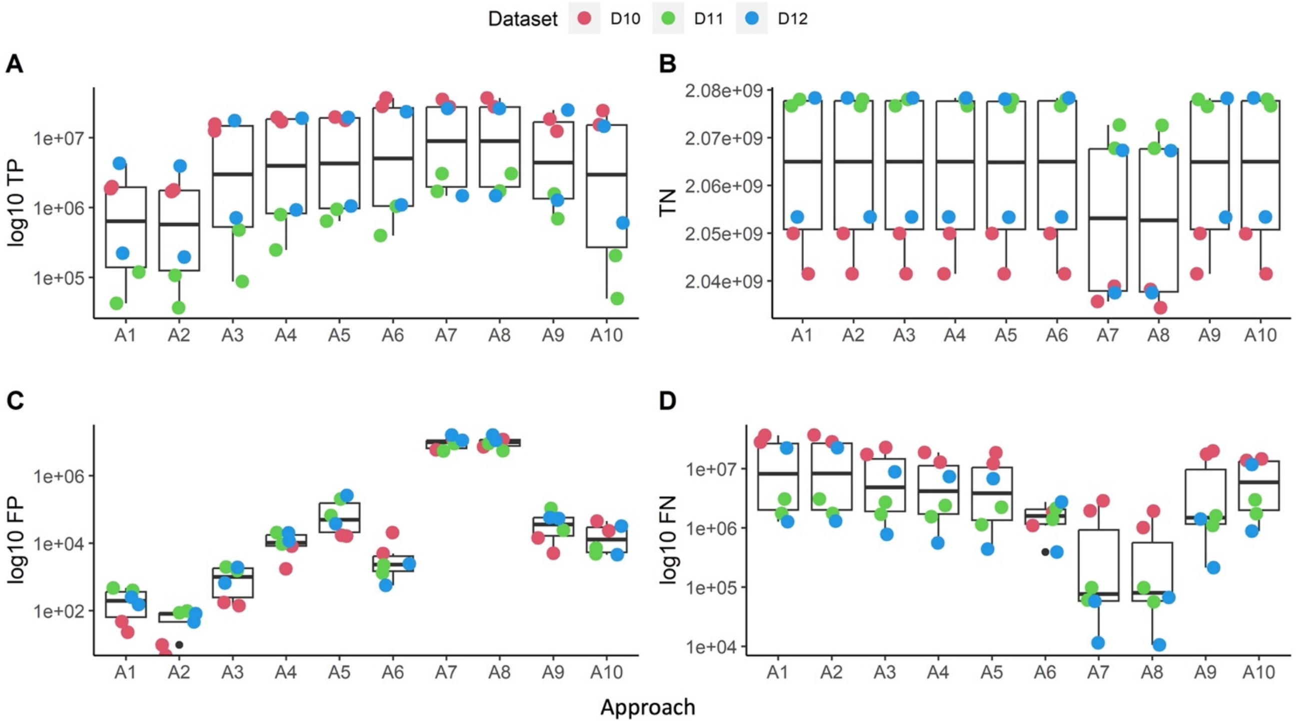

Supplementary Figure 7. Normalized number of TP, TN, FP, and FN cases produced by the ten approaches when applied to six samples from three simulated datasets (D10, D11, D12). Cases are normalized w.r.t. the largest total number of cases (i.e., sequence pairs; 2, 079, 834, 760) of dataset D11 (sample 1). That is TP (Sx, Dy) = TP (Sx, Dy) * Total number of cases Sx (Dy) / 2, 079, 834, 760. Here Sx represents a specific sample from dataset Dy.
